# Novel Thermal and Microscopic Techniques To Determine the Causes of Suboptimal Combustion Performance at Colombian Stoker Furnaces

**DOI:** 10.1021/acsomega.1c06314

**Published:** 2022-04-02

**Authors:** Orla Sioned Aine Williams, Patrick Daley, Joseph Perkins, Shoaib Shah, Edward Andres Garcia Saavedra, Maria Trujillo, Juan Barraza-Burgos, Carlos Julio Espitia, Maribel Barajas, Juan Sebastian Saltaren, Nicolás Javier Gil, Edward Henry Lester

**Affiliations:** † Faculty of Engineering, University of Nottingham, University Park, Nottingham NG7 2RD, U.K.; ‡ Mineral Resources, Commonwealth Scientific and Industrial Research Organisation, 1 Technology Court, Pullenvale, QLD 4069, Australia; § Facultad de Ingeniería, Universidad Del Valle, Ciudad Universitaria Meléndez, Calle 13 # 100-00. A. A., Cali 439, Colombia; ∥ Servicio Geológico Colombiano, Diagonal 53 No. 34−53, Bogotá D.C. 11121, Colombia; ⊥ Grupo Manuelita, Calle 6 # 3−13, Cali 760044, Colombia; # Centro de Investigación de la Caña de Azúcar de Colombia, Programa de procesos de fábrica, calle 58 Norte No 3BN. 110, Cali 780001, Colombia

## Abstract

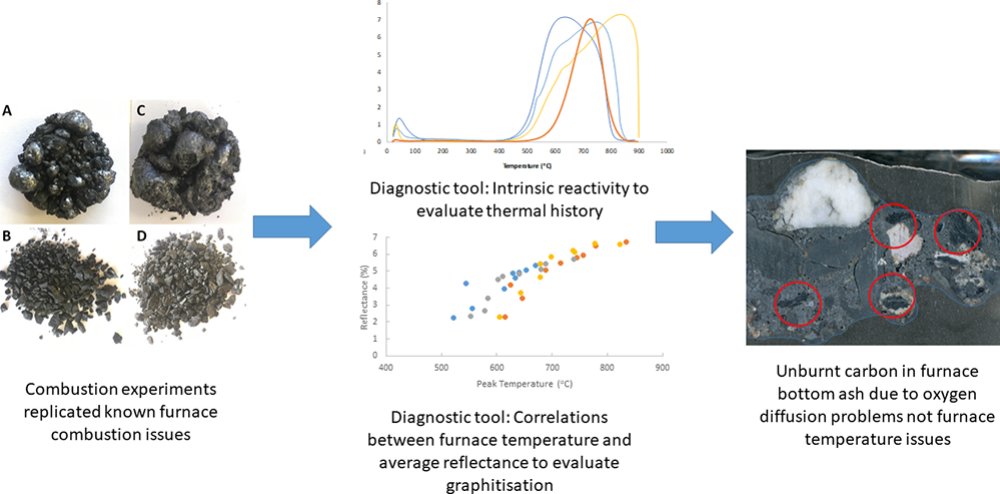

This study presents the application of a novel approach, using thermal and optical techniques, to identify the causes of poor burnout performance of Colombian stoker furnaces in the Cauca Valley State. The four coals used in these furnaces were characterized to obtain particle size distribution, particle and tapped density, elemental and proximate composition, mineral composition, and maceral content. Up to 80% incomplete combustion was noted in macro-TGA tests compared to complete combustion in a micro-TGA. Reflectance and intrinsic reactivity measurements were for chars prepared in three different particle sizes (<6, 6–19, and 19 mm), three temperatures (700, 900, and 1050 °C), and three residence times (10, 30, and 120 min). Two of the coals produced char samples with reflectance values above 6%, which matched those seen in the stoker, indicating that the furnace temperature was not the cause of poor combustion and that only two of the four coals were likely to be present in the furnace bottom ash. These tests were also able to prove that oxygen diffusion limitation was the root cause of the poor burnout where the carbon inside the furnace bottom ash was shielded from oxygen ingress through the formation of a nonpermeable slag layer. Thus, this study demonstrates the potential of both thermal profiling and optical reflectance as a tool for forensically evaluating the thermal history and operational performance of furnaces.

## 1 Introduction

The origins of incomplete combustion of fuels in stoker furnaces can be complicated to diagnose, but they normally result from either fuel selection or furnace operation.^1−3^ Stoker furnaces, or traveling grate spreader stoker furnaces, are widely used by the pulp and paper, sugar, and cement industries and also generate electricity to meet national demand in countries such as South Africa, Namibia, and Colombia.^3,4^ While pulverized fuel (normally below 212 microns) is expected to burn out in less than 2 s,^5^ stoker furnaces use coal particles in the size range of 6–25 mm with residence times of 15 min up to several hours.^3,6−8^ It can be difficult to optimize stoker furnace performance and identify the source of combustion-related issues.^3,9^ The novel application of fuel characterization techniques could help boiler operators tackle these issues.

Furnace bottom ash (FBA) represents up to 90% of the ash residue from stoker furnaces.^10^ Unburnt carbon in FBA can be a common problem.^11−15^ Fly ash generally consists of solid spheres and hollow spheres (cenospheres), and unburned carbon is directly related to the partial burnout of coal particles.^16^ Fly ash particles are generally below 200 μm^12,17^ and 1440 μm for biomass.^18^ In contrast, stoker FBA is generally nonspherical with a particle range of 2 μm to 20 mm, specific gravity between 1.5 and 2.8, and bulk density between 700 and 1600 kg/m^3^.^19^ It has been shown for municipal solid waste combustion on fixed bed reactors that increasing ash content results in a lower fuel burning rate.^20^ Slow burnout can lead to incomplete combustion and a reduced combustion efficiency. While there is a significant body of research into unburnt carbon and incomplete combustion in pulverized fuel (PF) power stations,^15,21,22^ there is limited information on the causes of incomplete combustion of coals in stoker furnaces. Furthermore, few analytical characterization techniques have been applied to stoker furnace fuels. Stoker furnace coals are normally of lower quality than PF coals, and their size poses challenges in the application of these techniques.

Incomplete combustion can often be caused by poor coal quality, which can, in turn, be linked to proximate and ultimate analysis and petrographic properties such as maceral composition and maceral reflectance. Reflectance provides an indicator of coal rank and is defined as the proportion of normally incident light that is reflected by a plane, polish surface of the coal under analysis.^23^ Reflectance parameters illustrate the average degree of three-dimensional ordering of the molecular structure of organic constituents.^24^ Normally, vitrinite reflectance is the standard used when measuring the coal rank, but the coal loses its maceral “identity” as it moves toward a more ordered graphitic structure. However, this optical reflectance can relate to all stages of crystallinity ranging from amorphous to graphitic forms from biomass through to graphite.^25^ The crystal structure causes directional variations in the transmission or reflection of polarized light, which provide the reflectance values.^26^ Clearly, reflectance increases during combustion, and higher maximum reflectance values indicate a more ordered carbon molecular structure.^27^ Reflectance is also commonly used to establish coke quality^28^ because there is a direct link between the reflectance of the coke and the furnace conditions used, as well as coal maturity.^29^ Charcoal reflectance was shown to be a way to determine the regional intensity of wildfires^30^ where reflectance levels were found to be the greatest in areas that had experienced the most severe fires.^31^ Furthermore, reflectance from forest fire chars was used to determine the type of fire and the fuel that it originated from.^32^ However, no studies to date have used reflectance to analyze the thermal history of a stoker furnace.

This paper is a detailed investigation into the causes of suboptimal burnout performance, and the causes of incomplete burnout are identified through the characterization of the fuels along with the fly ash and FBA. To date, char reflectance and intrinsic reactivity profiling has not been used to assess the thermal history of a stoker furnace ash, and this study presents the first use of these methods to identify a key issue affecting the furnace performance of stoker furnaces in Colombia.

## 2 Results and Discussion

### 2.1 Overview of the Combustion Issues Found in Colombian Stoker Furnaces

Incomplete combustion was experienced in the Colombian stoker furnaces when the four coals in this study were used as a fuel. Carbon-in-ash values from the FBA were found to be as high as 24%, which is surprisingly high for a furnace operating at ∼900–1000 °C with a residence time of several hours.

The possible causes for this were identified as follows:

i. Unreactive coals—coals or blends of coals that have a lower reactivity (e.g., anthracite or artificially oxidized coals) or contaminated with unreactive materials such as metallurgical or petroleum coke.
ii. Combustion temperatures—while online camera systems measure surface temperature, it might be possible that internal bed temperatures are not operating as high as expected.
iii. Oxygen levels—either through poor control of overfire air or oxygen diffusion in the stoker bed, meaning that there was not sufficient oxygen to ensure complete combustion.
iv. Ash issues—caused by the presence of high levels of ash in some of the coals.
v. Potentially a combination of all four issues.

### 2.2 Coal Characterization

The target particle size specification for stoker furnaces is generally 6–25 mm because <6 mm can fall through the grate during combustion and >25 mm can potentially damage screw feeders.^3^Table 1 shows that the coals provided for this study were found to have large amounts of material above and below the target size range. In some cases, less than 30% of the sample was within the desired 6–19 mm particle size range e.g., 55% of Patia and Antioquia, and 43% of Valle and 19% of Cundinamarca would be classified as fines (<6 mm). Apart from potential losses under the grate, excessive fines can magnify coal segregation problems and an increased packing density can also lead to problems with incomplete combustion on the grate.^33^ Fines can restrict the under-fire air penetrating the bed and can lead to clinker formation and lower grate temperatures. In addition, Cundinamarca had 47% oversize material (>19 mm) including some very large pieces over 50 mm.

**Table 1:** Particle Size Distribution for Patia, Valle, Cundinamarca, and Antioquia Coals

size	Antioquia	Cundinamarca	Patia	Valle
under size (<6 mm), %	55	19	55	43
within specification (6–19 mm), %	21	27	25	27
over size (>19 mm), %	24	47	20	30

Due to a significant portion of each coal being undersize or oversize, the composition of the coal was analyzed by particle size where possible to examine the influence of particle size on incomplete combustion. Table 2 shows the proximate analysis and density measurements for each coal. Apart from Valle, the coals have significantly more ash in the fine fraction compared to the target size range and oversize. Antioquia has the lowest ash in the oversize (4.5%), whereas the fines contained more than three times as much (13%). Patia had almost four times as much as ash in the 6–19 mm (21.2%) and fine (26.5%) size ranges compared to the oversize (6.2%). Cundinamarca showed the least variability across the three size ranges (11.2–16.9%). Valle showed a different trend to the other coals, with the oversize having the largest amount of ash (37.3%) with the lowest value in the “fines” (29.2%). In all sizes, Valle had significantly more ash than the other three coals. The bulk and tapped density of all the coals was similar. Valle fines had the highest particle density (1.57 g/cm^3^) and Cundinamarca the lowest (1.39 g/cm^3^). Buoyancy density varied significantly with Valle showing the lowest value (1.14 g/cm^3^) and Antioquia the highest (1.59 g/cm^3^). Tap density for all four coals was relatively similar.

**Table 2:** Proximate Analysis and Density Measurements for the Coals

sample	size (mm)	moisture (%)	dry volatile matter (%)	fixed carbon (%)	dry ash (%)	bulk density (g/cm^3^)	tapped density (g/cm^3^)	particle density (g/cm^3^)	buoyancy density (g/cm^3^)
Antioquia	<6	7.8	41.9	45.1	13.0	0.79	0.89	1.40	
	6–19	8.0	44.6	47.1	8.3	0.64	0.67		1.59
	>19	9.1	49.0	46.5	4.5				
Cundinamarca	<6	1.9	34.9	48.1	16.9	0.71	0.82	1.39	
	6–19	2.1	35.7	50.2	14.1	0.57	0.62		1.27
	>19	1.6	37.4	51.4	11.2				
Patia	<6	4.6	34.9	38.6	26.5	0.76	0.86	1.47	
	6–19	4.2	36.8	42.0	21.2	0.56	0.64		1.37
	>19	4.4	46.7	47.1	6.2				
Valle	<6	1.6	29.2	41.5	29.2	0.76	0.84	1.57	
	6–19	1.1	27.7	39.0	33.3	0.62	0.68		1.14
	>19	1.0	36.4	26.2	37.3				

All the coals in this study are within the expected range for elemental composition for coals.^34^ The differences in C and O are the greatest in lower rank coals, i.e., Patia and Antioquia. The differences in N and S, however, appear to remain essentially the same, regardless of the coal rank. From the data in Tables 2 and 3, there is nothing to suggest that any of these coals would give poor combustion.

**Table 3:** Elemental and Petrographic Characteristics of the Bulk Coals

parameters	Antioquia	Cundinamarca	Patia	Valle
carbon (%)	56.5	66.1	47.6	52.2
hydrogen (%)	4.9	4.8	4.0	3.9
nitrogen (%)	1.4	1.6	1.3	1.1
sulfur (%)	0.9	1.1	1.3	2.5
gross dry higher heating value (J/g)	25,333	29,945	24,532	21,948
dry ash-free higher heating value (J/g)	28,139	34,138	31,098	32,630

### 2.3 Petrographic Analysis

The maceral and vitrinite reflectance of the coals are shown in Table 4 for the three size fractions. These coals are known to be sourced from regions rather than individual mines and are therefore potentially from multiple seams from across that region.

**Table 4:** Petrographic Analysis of the Four Coals in Their Respective Size Fractions

maceral analysis												
	Antioquia			Cundinamarca			Patia			Valle		
	<6 mm	6–19 mm	>19 mm	<6 mm	6–19 mm	>19 mm	<6 mm	6–19 mm	>19 mm	<6 mm	6–19 mm	>19 mm
vitrinite	95.4	96.0	99.0	90.0	77.6	36.0	99.0	97.0	91.0	99.2	99.4	97.6
liptinite	0.0	0.4	0.0	0.4	3.0	2.0	0.4	0.0	2.4	0.2	0.0	0.4
semifusinite	4.0	3.0	0.6	4.0	11.0	44.0	0.0	3.0	4.6	0.4	0.6	1.2
fusinite	0.6	0.6	0.4	5.6	8.4	18.0	0.6	0.0	2.0	0.2	0.0	0.8
vitrinite reflectance analysis												
	Antioquia			Cundinamarca			Patia			Valle		
	<6 mm	6–19 mm	>19 mm	<6 mm	6–19 mm	>19 mm	<6 mm	6–19 mm	>19 mm	<6 mm	6–19 mm	>19 mm
average	0.43	0.43	0.36	0.68	0.90	0.68	0.57	0.53	0.48	0.95	0.62	0.73
minimum	0.37	0.39	0.26	0.43	0.47	0.48	0.43	0.41	0.35	0.49	0.48	0.44
maximum	0.50	0.51	0.44	1.04	2.19	1.14	0.73	0.66	0.65	1.53	0.84	1.39
SD	0.03	0.03	0.05	0.164	0.218	0.113	0.08	0.05	0.07	0.28	0.06	0.17

In most cases, the coal samples have a high vitrinite content apart from Cundinamarca where the vitrinite is very low in the >19 mm (<40%). The implication here is that there are more than one petrographic composition coming from the Cundinamarca region with distinctly different maceral compositions.

The vitrinite reflectance data in Table 4 also show significant variation between coals (0.4% for Antioquia to 1.0% for Valle) as well as variability between size fractions, particularly with Valle and Cundinamarca. Analysis of the vitrinite reflectance profiles (Figure S1A–D) indicates that all Valle and Cundinamarca are blends of quite different coal types. The explanation as to the variance in the different size fractions could be explained by the grading of the coals from each region. The 6–19 mm fraction for Valle shows only lower reflectance material, whereas the finest fraction is probably a mix of at least three coal seams, with parts of the coal exhibiting very high and low vitrinite reflectance in addition to medium rank portions. Cundinamarca shows a similar divergence. The 6–19 mm fraction is mainly higher reflectance coal (0.7–1.2%), whereas the <6 mm fraction is mainly lower reflectance (0.4–0.8%). The >19 mm fraction is a mixture of both higher and lower reflectance coal.

The specific regions of Cundinamarca and Valle that produce the highest reflectance coals into the blended coal could consider selling these seams as coking coals, as they carry a higher premium over coals for combustion. Vitrinite with a reflectance higher than V7 forms anisotropic structures. The highest reflectance fraction from Valle would generate lenticular to ribbon structures based on the vitrinite reflectance range V12–13.^25^ However, it is important to note that despite being blends across a relatively high reflectance range (in some cases), all four coals are suitable for combustion, particularly in a stoker furnace where residence times are hours rather than seconds.

Essentially, there is nothing obvious in the petrographic composition to explain the cause of the high carbon in ash and nonoptimal burnout.

### 2.4 Coal Reactivity and Burnout Rates

**Figure 1:**
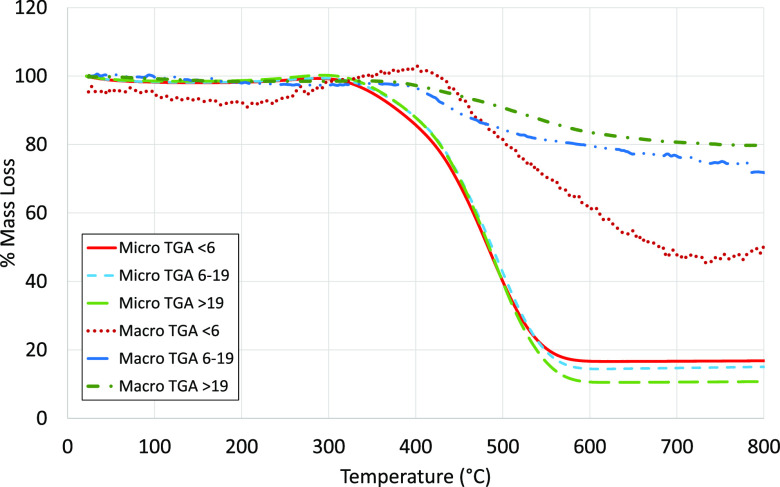
Macro- and micro-TGA profiles for all size fractions of Cundinamarca.

Tests were carried out to measure the combustion rates of each coal using a micro- (with milled coal) and macro-TGA (unmilled). Figure 1 shows the comparative mass loss for Cundinamarca in the macro- and micro-TGAs by a particle size group. The figures for Valle, Antioquia, and Patia can be found in Supplementary Figure S2A–C. The micro-TGA burnout rates are similar for all particle size groups showing a clear onset of burnout and steady states beyond 600 °C where combustion has been completed. While complete combustion was achieved in the micro-TGA, it was clear that incomplete combustion was occurring in the stoker furnace. The aim of the macro-TGA was to replicate this incomplete combustion. Figure 1 illustrates that incomplete combustion was replicated in the macro-TGA. Final weight loss was around 50% for <6 mm samples, 70% for 6–19 mm, and 80% for >19 mm. For the macro-TGA, a very slight weight gain can be noted for the <6 mm sample, which is a result of changes in buoyancy due to its lower mass compared to the larger samples (∼1 g).^35^ Above 600 °C, below 0.5 g of the sample remained, which resulted in it being sensitive to any fluctuations, as the linearity deviation of the balance is up to 0.2 g. Furthermore, Cundinamarca and Valle were known to swell during combustion, which is discussed further in Section 4 with reference to the literature.^36^ This explosive swelling behavior also led to fluctuations in the mass recorded during the tests.

Table 5 shows the breakdown of intrinsic reactivity data for both micro- and macro-TGA by size and final mass. For Cundinamarca, combustion begins around 289 ± 6 °C for all particle sizes, with the peak burnout at 490 ± 4 °C and final burnout at 593 ± 5 °C. Valle was clearly the least reactive with an initial combustion temperature of 305 ± 5 °C, peak burnout at 506 ± 2 °C, and final burnout at 602 ± 5 °C. Overall reactivity ordering shows Antioquia > Patia > Cundinamarca > Valle, which is a trend linked to the petrographic composition and vitrinite reflectance values in Table 4.

**Table 5:** Initial Combustion Temperature (*T*_i_), Peak Burnout Temperature (*T*_p_), Final Burnout Temperature (*T*_b_), and Final Mass (*M*_f_) from Micro- and Macro-TGA Tests

sample	size (mm)	micro-TGA				macro-TGA			
		*T*_i_ (°C)	*T*_p_ (°C)	*T*_b_ (°C)	*M*_f_ (%)	*T*_i_ (°C)	*T*_p_ (°C)	*T*_b_ (°C)	*M*_f_ (%)
Antioquia	<6	258	402	509	11.7	335	469	638	63.4
	6–19	265	432	538	7.6	362	466	702	21.1
	>19	259	449	523	4.2	362	506	743	20.4
Cundinamarca	<6	287	486	590	16.5	417	533	709	40.2
	6–19	294	494	595	14.3	377	447	601	71.9
	>19	285	489	593	10.0	342	527	718	79.4
Patia	<6	272	463	538	25.4	271	445	597	63.0
	6–19	273	458	545	20.0	371	446	613	47.2
	>19	267	441	542	5.7	405	472	616	52.8
Valle	<6	301	505	597	28.8	454	508	691	56.9
	6–19	305	505	601	33.2	396	518	729	61.9
	>19	308	509	607	37.0	382	470	741	53.8

In all cases, the micro-TGA runs show complete combustion where the wt % loss matches the ash % from proximate analysis.

The most significant finding is that the rate of combustion once started is slower and the final burnout levels are much lower for the macro-TGA system. Combustion does not appear to completely stop in all cases but is very slow implying inhibited combustion; while air ingress onto the samples (for both micro- and the macro-TGA technique) is from above, the most significant difference is that the size of the sample in the macro-TGA is 10 g rather than 10–20 mg. The samples still have access to air, so the larger sample size should not be an issue, particularly as furnace temperatures reach in excess of 700–800 °C. For more accurate results, the macro-TGA system would need further refinement, but this study proved in principle that the macro-TGA can replicate incomplete combustion on a laboratory scale and that the mass loss was significantly different to that obtained in a micro-TGA.

### 2.5 Devolatilization Samples

**Figure 2:**
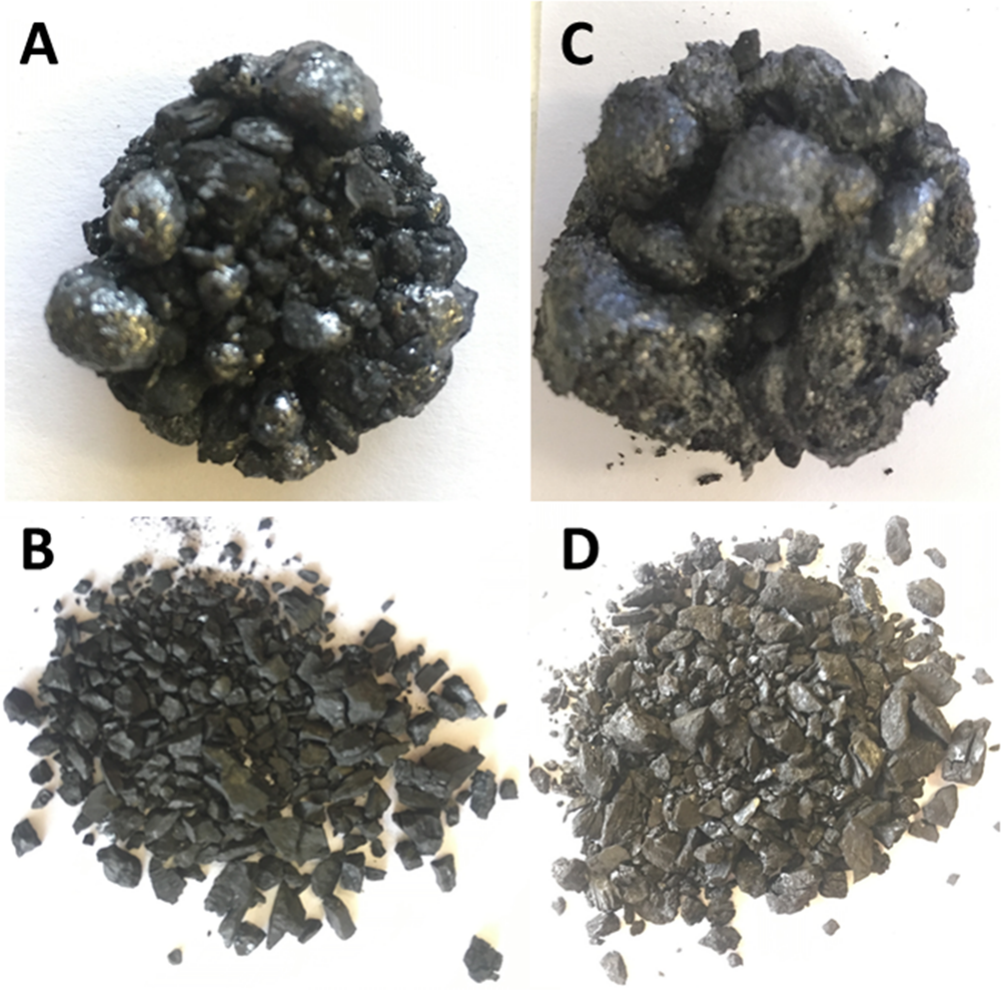
(A) <6 mm Cundinamarca and (B) Valle fused chars, and (C) Antioquia and (D) Patia loose chars produced after 10 min at 700 °C in an inert atmosphere in a muffle furnace.

A series of experiments was carried out to see if the coals themselves were able to fully devolatilize when held at fixed temperatures and times characteristic of a stoker furnace. Each coal was heated in three particle sizes (<6, 6–19, and >19 mm) to three temperatures (700, 900, and 1050 °C) for three residence times (10, 30, and 120 min) in an inert atmosphere (nitrogen). It was noted that two of the samples, Valle and Cundinamarca, tended to swell and fuse during the muffle heating process at all temperatures and residence times (Figure 2), while Patia and Antioquia did not fuse.

**Figure 3:**
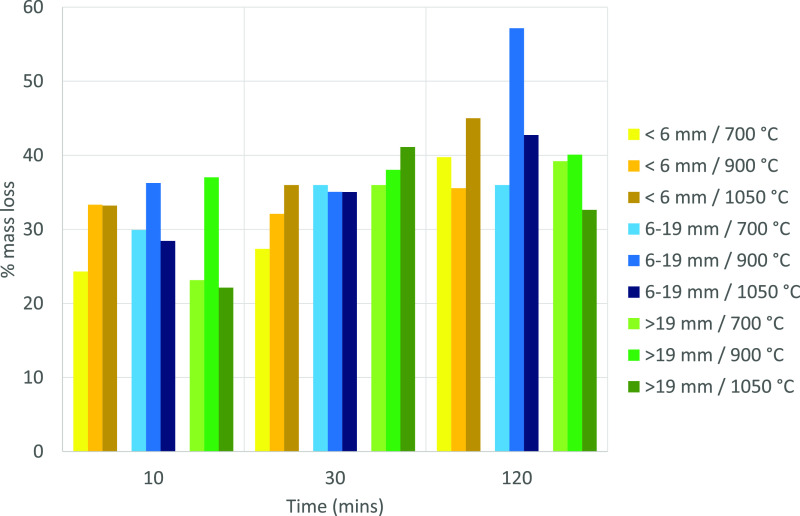
Mass loss in the muffle furnace for varying particle sizes, temperatures, and residence times for Cundinamarca coal.

Figure 3 shows the weight loss for Cundinamarca. Comparing results to proximate analysis data (Table 2), these muffle furnace “chars” all show that ∼90% of their volatiles were removed after 10 min. The results for the other coal samples are shown in Supplementary Section Figure S3. In all cases, there was little difference between 10, 30, and 120 min samples, and regardless of the mechanism that retarded combustion in the macro-TGA, devolatilization was relatively rapid relative to the 2–5 h available in the actual Stoker furnace.

As such, it is unlikely that the poor combustion results seen in the macro-TGA were related to the inhibition of the devolatilization rates of the coals.

### 2.6 Temperature Profiling Using Char Reflectance

Another possible reason for the slower combustion rates seen in the macro-TGA and the poor burnout rates seen in the stoker furnace is a lower bed temperature (in the carbon on the bed) resulting in thermal losses from the ash layers.

**Figure 4:**
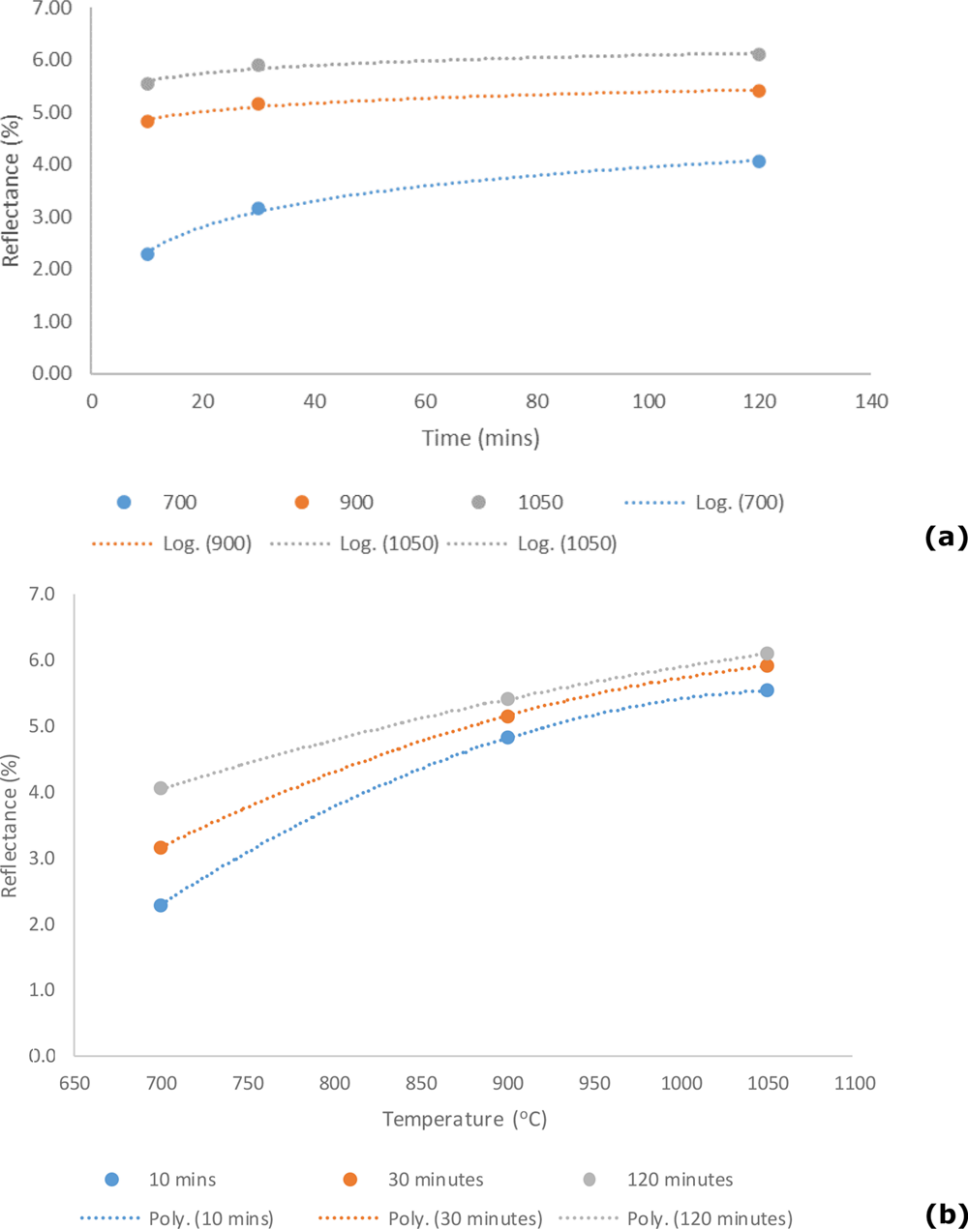
(a) Influence of residence time on coal reflectance for varying residence times and (b) influence of furnace temperature on coal reflectance for varying furnace temperatures.

The reflectance of the samples generated in Section 2.5 was measured to establish a link between reflectance and time/temperature. Reflectance is linked to C/H ratios, which changes predictably during the combustion process,^37^ and so, it is logical that as time increases so does the graphitization process, which leads to an increase in the reflectance of the unburned carbon.^38−40^ The reflectance of the samples from all four coal types for all particle sizes was averaged for residence time and furnace temperature (Figure 4a,b). Figure 4a shows a correlation between the change in reflectance with residence time although the majority of the change in reflectance (from the original coals at <1% reflectance) has clearly occurred in the first 10 min. However, residence time was less significant as residence time increases, particularly at 900 and 1050 °C.

Figure 4b illustrates a strong linear correlation between average reflectance and furnace temperature. Higher temperatures result in a significant increase in reflectance. However, it is clear that average reflectance for the three residence times converges as temperature increases.

Figure S4 shows the specific profiles for each of the coal samples individually for reflectance vs temperature and Figure S5 for reflectance vs time. The trends can be modeled using power law and extrapolated to 9 h, which is twice as long as the residence times seen in the actual stoker. Table S1A–D shows the detailed reflectance results, including minimum and maximum reflectance and standard deviation for all 108 samples. The 700 °C results generally show the largest standard deviations, which indicates a lack of uniformity (or the largest range of graphitization) in the reflectance values. Standard deviation decreases with the increasing residence time and increasing temperature, which indicates that the carbon starts to converge on a similar degree of carbonization over time. Table 6 shows the reflectance values for each coal type extrapolated to 9 h. This was done to see that all four coals were capable of producing reflectance levels similar to those seen in the FBA char if they are given enough time, albeit 2× as long as the actual residence times in either stoker furnaces.

**Table 6:** Average Reflectance Values for the Samples from Each Coal Type with Time and Temperature

	residence time (min)	temperature (°C)		
		700	900	1050
Antioquia	10	2.23	4.28	4.92
	30	2.82	4.59	5.08
	120	3.96	4.86	5.34
	540	5.57	5.27	5.61
Cundinamarca	10	2.31	5.05	5.94
	30	3.43	5.50	6.51
	120	4.18	5.79	6.73
	540	5.71	6.34	7.33
Patia	10	2.33	4.51	5.11
	30	2.69	4.68	5.43
	120	3.42	4.83	5.71
	540	4.28	5.04	6.12
Valle	10	2.28	5.44	6.21
	30	3.71	5.86	6.62
	120	4.65	6.15	6.61
	540	6.18	6.66	6.93

**Figure 5:**
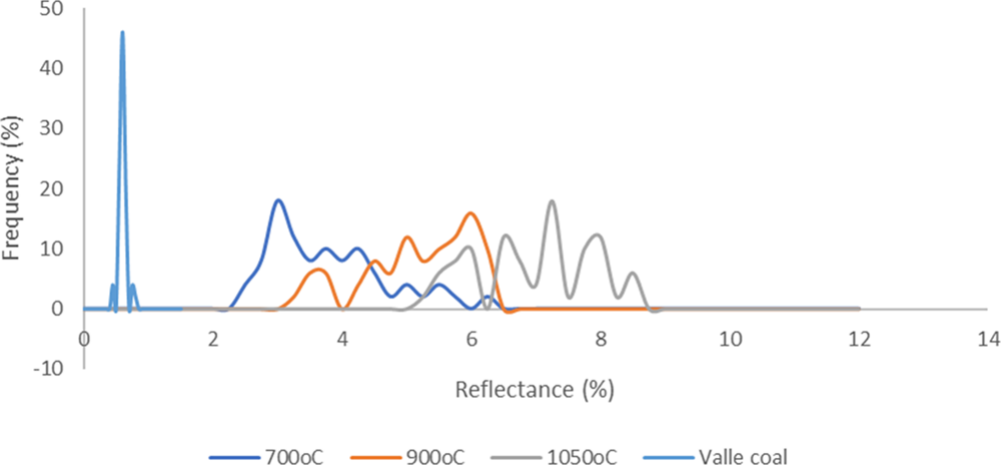
Reflectance for Valle at 700, 900, and 1050 °C for a residence time of 120 min.

Figure 5 shows the reflectance profiles of Valle at the three different temperatures for a residence time of 120 min. Clearly reflectance increases with the increasing temperature with some material having a reflectance >8%, which is similar to the reflectance of high-grade coke.

These reflectance values for each sample can also be correlated with the values for the carbon found in the Colombian stoker boilers. Table 7 shows the reflectance data for chars and both fly ash and FBA from La Cabaña and Mayagüez. Values for the FBA and fly ash are above 6.0%, indicating that high temperatures have been achieved.

**Table 7:** Reflectance of Char, Furnace Bottom Ash and Fly Ash from the La Cabaña Sugar Mill and Furnace Bottom Ash and Fly Ash from the Mayagüez Sugar Mill

	La Cabaña furnace bottom ash	La Cabaña char 1	La Cabaña char 2	La Cabaña fly ash	Mayagüez furnace bottom ash	Mayagüez fly ash
average	6.50	6.02	6.69	6.55	6.09	6.20
minimum	4.98	4.15	5.12	4.62	3.73	4.39
maximum	10.06	7.96	8.08	8.66	9.43	8.83
SD	1.203	0.855	0.838	1.003	1.468	1.261

**Figure 6:**
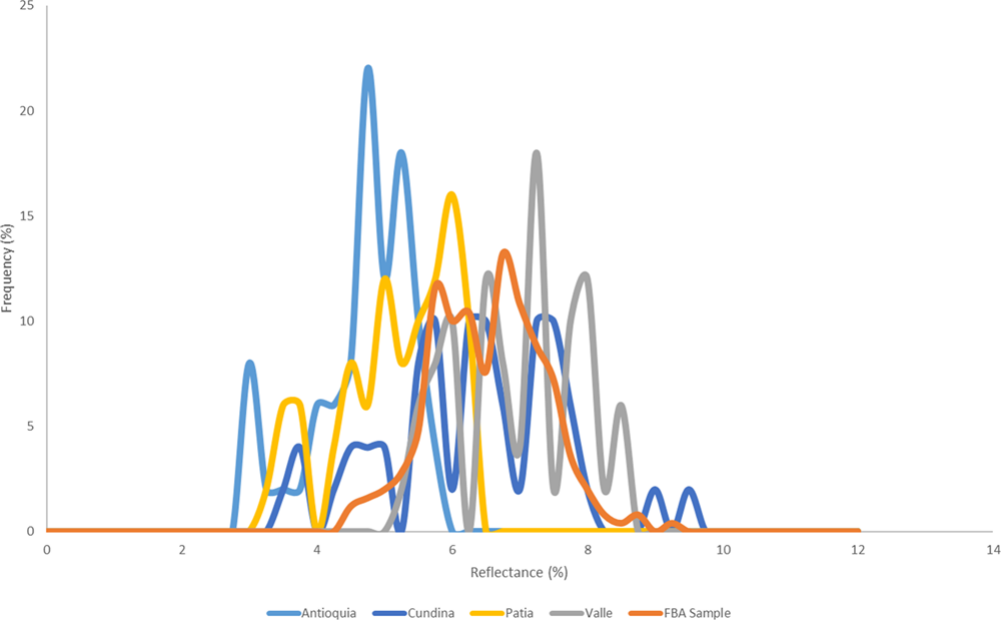
Reflectance profiles for chars from the four coals created at 1050 °C and 120 min compared with La Cabaña furnace bottom ash (<6 mm).

Figure 6 shows the reflectance profiles at 120 min at 1050 °C from all four coal samples compared to the reflectance profile from the carbon found in La Cabaña sample 1. In this case, the closest match is with Valle and Cundinamarca. Patia and Antioquia both have a significantly lower reflectance range at 2.7–6% and 3.0–6.5%, respectively.

The average values at each temperature and time correlates with the average reflectance range of the carbon samples found in the FBA/fly ash. Patia and Antioquia did not appear to be able to create average reflectance values above 6%. Even after extrapolation of 9 h at 1050 °C (Table 2), Antioquia can only generate 5.6% and Patia 6.1%, which does not map over the reflectance ranges seen with the FBA samples.

Industrial furnaces operating at 900–1000 °C have produced a reflectance of 6.0–6.5%. A temperature of 900–1050 °C was required to create a reflectance level of >6% in the muffle furnace, and Cundinamarca and Valle are the most likely to form carbon with reflectance >6%. These experiments also prove that the bed temperature is unlikely to be the issue, which might have been arisen from heat losses caused by high ash content coals.

### 2.7 Intrinsic Reactivity Profiles

**Figure 7:**
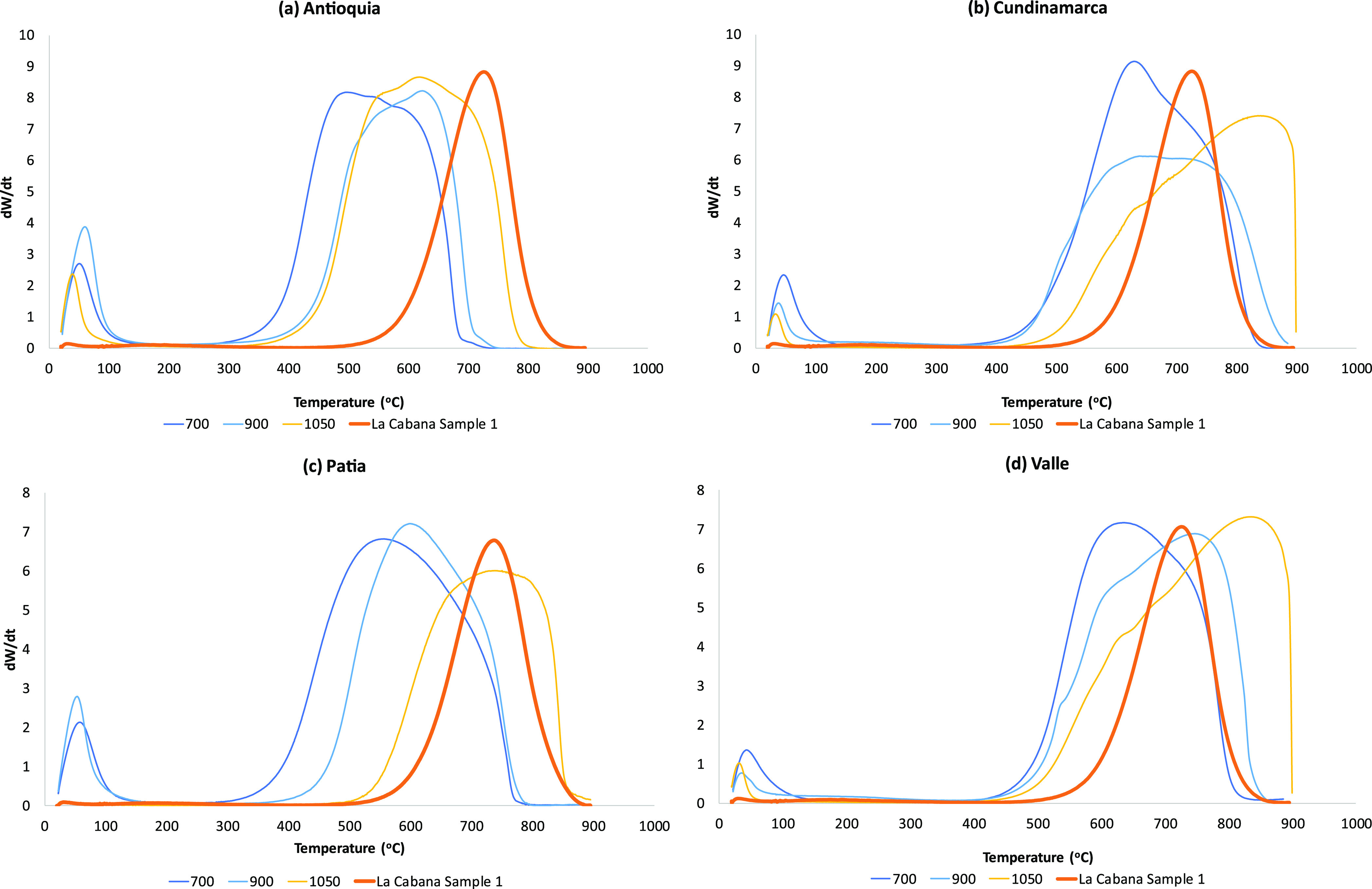
Intrinsic reactivity profiles for the char samples prepared at 700, 900, and 1050 °C (120 min) vs the La Cabaña FBA sample for (a) Antioquia, (b) Cundinamarca, (c) Patia, and (d) Valle.

Figure 7 shows the intrinsic reactivity profiles for chars from each coal type prepared at 700, 900, and 1050 °C at 120 min compared to an FBA profile for La Cabaña. The position of the peaks is a key indicator of reactivity^28^ where the further to the right the peak is, the less reactive the carbon is. Unsurprisingly, the profiles from chars formed at 700 °C are more reactive than those formed at 900 or 1050 °C. The carbon present in the FBA is also relatively unreactive with a peak temperature around 730 °C. Antioquia (even at 1050 °C) does not produce a burnout profile that matches that of the FBA profile. Only the 1050 °C sample of Patia char covers the same range as the FBA sample, where Valle and Cundinamarca both produce burnout profiles at 900 °C that span the same temperatures. At 1050 °C, both Valle and Cundinamarca produce even less reactive chars than those found in the FBA.

**Figure 8:**
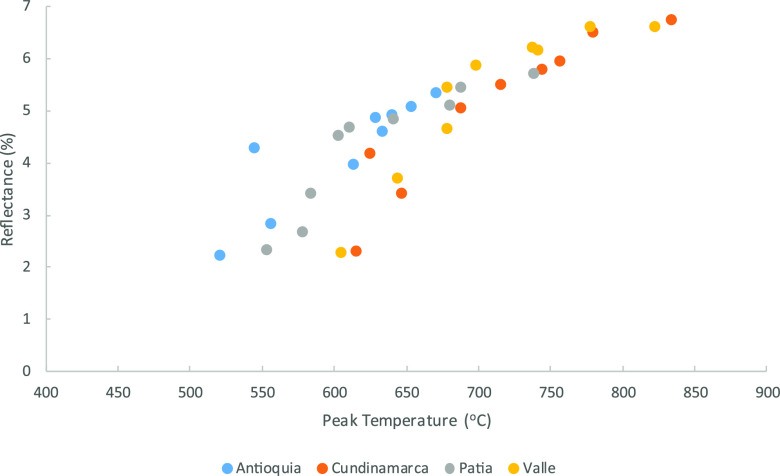
Correlation between peak temperature (from intrinsic reactivity) and average reflectance.

The full set of intrinsic data (including initial, peak, and burnout temperatures) are given in Supplementary Table S2A–C for all 108 samples. The correlation between reflectance and peak temperature is shown in Figure 8. Clearly, there is a general trend with some scatter, which is caused by the heterogeneous nature of the original coals, in terms of maceral composition. The graphitization rate is different for different macerals, and Cundinamarca has the highest levels of inertinite, which is known to be more reluctant to structurally reorder during heating.^41^

In a similar way to the reflectance measurements in Section 2.6, Valle and Cundinamarca produce results that are closest match to the values seen in the FBA and fly ash samples.

### 2.8 Influence of Minerals on Combustion Behavior

The relatively high ash content of some of the coals was a key characteristic for these coals and potentially a reason for incomplete combustion. From Sections 2.5 and 2.6, it is clear that the ash in the coal did not inhibit devolatilization or lower bed temperatures, but the overall findings in Sections 2.6 and 2.7 point to Cundinamarca and Valle as being the most likely to produce the type of carbon material found in the FBA. Antioquia and Patia appear to be the least likely as they are not able to produce carbon with the same reflectance or intrinsic reactivity.

**Figure 9:**
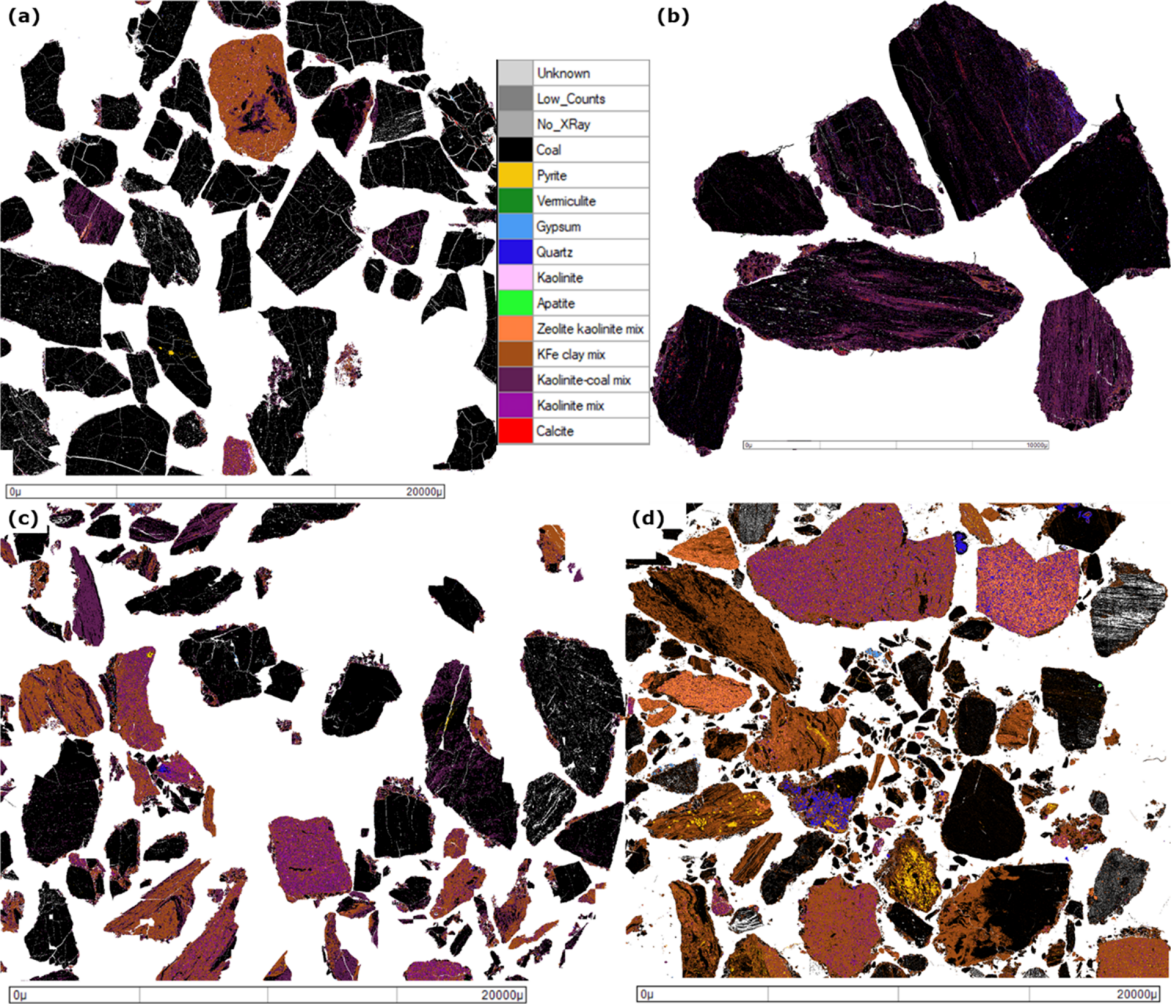
MLA analysis of the −6 mm fractions of (A) Antioquia, (B) Cundinamarca, (C) Patia, and (D) Valle.

Mineral liberation analysis (MLA) was used to map the composition and textural characteristics of each coal. Figure 9A–D shows the mineral composition for the <6 mm fraction for the four coals. Images can be found in Supplementary Section Figure S6A–D for the 6–19 mm and S7A–D for the >19 mm size fractions. Variants of Kaolinite were the most commonly identified minerals, which is a clay predominantly composed of aluminum silicates (chemical composition Al_2_Si_2_O_5_(OH)_4_). Antioquia had the lowest ash content (13%) as can be seen in Figure 9A, which shows Antioquia to be composed of mainly coal particles or coal particles with small ribbons of finely dispersed kaolinite. Cundinamarca is mainly coal with larger kaolinite ribbons.

Table S4 shows that Valle and Patia (Table S3 contains the data for Antioquia and Cundinamarca) both show significantly higher ash contents with the −6 mm fractions, at 29.2 and 26.5%, respectively, based on MLA. Valle, in particular, shows a much lower amount of coal only particles (<10% in the largest size fraction) and is mainly a combination of discrete kaolinite-based particles or different minerals intimately associated with the coal inside each particle. This level of inherent mineral (within the coal particles) in Valle means that liberation of the coal from the mineral matter depends directly on particle size. With the mineral matter in the coal matrix, the coal becomes liberated in the finer fractions.^42^ In this case, the −6 mm material shows an increased level of liberation (50% instead of 6%), but this is technically out of the size range specified for use in the Stoker furnace. Based on the proximate analysis in Table 2 (that shows a high ash content in the −6 mm size fraction) and the MLA data, it is clear that most of the mineral matter in Patia is already liberated into the fines.

X-ray fluorescence (XRF) data are given in Table S5 and show similar data to those of SiO_2_ and Al_2_O_3_ representing over 80% of the Colombian coals and FBA and fly ash samples. This originates from the kaolinite predominantly.

### 2.9 Oxygen Diffusion

Table 8 shows the initial deformation (IDT) and flow temperatures for the ash from each coal type and the fly ash and FBA samples from the two stoker furnaces.

**Table 8:** Ash Fusion Data for the Ash from the Coals and Stoker Furnace Samples

sample	Antioquia	Cundinamarca	Patia	Valle	La Cabaña FBA	La Cabaña fly ash	Mayagüez FBA	Mayagüez fly ash
initial deformation temp. (°C)	1250	1279	1410	1401	1211	1198	1324	1172
flow temp. (°C)	1322	1574	1583	1564	1520	1387	>1569	1321

The IDT values for all the coals are significantly higher than 1000 °C (mainly because of the large levels of Al_2_O_3_- and SiO_2_-based minerals), which indicates that the ash would not be able to flow in the stoker. However, while the ash material may not truly melt in the furnace, the high ash content of three of the four coals can still lead to poor oxygen diffusion. The global combustion rate of coal can be limited by the reaction kinetics of the char matter, the pore diffusion resistance of the char pore structure, and the bulk diffusive oxygen transport in the boundary layer of the particle.^43^ Char kinetic rates are dependent on particle temperature and oxygen concentration on the external particle surface.^44,45^ Sufficient oxygen needs to be transported to the coal surface for combustion to occur.

The minerals in the coals in this study are already present either in the form of liberated particles or intercalated layers that probably (once pyrolysis has finished and combustion has started) start aggregate together to form a barrier layer around any unburnt carbon.^46^ Current theories regarding the influence of ash on incomplete combustion for stoker furnaces and fluidized bed combustion are based on the “ash film” model.^47^ The theory is based on ash liberated during the combustion of the coal accumulating on the particle surface, which then hinders combustion by creating a diffusional barrier to the transport of oxygen to the encapsulated char inside the particle. Furthermore, the ash in all four coals appears to contain significant quantities of Al_2_O_3_ (>50 wt %), which can distribute evenly on the surface of coal particles during heating and form a dense protective film to hinder oxygen diffusion.^48^

**Figure 10:**
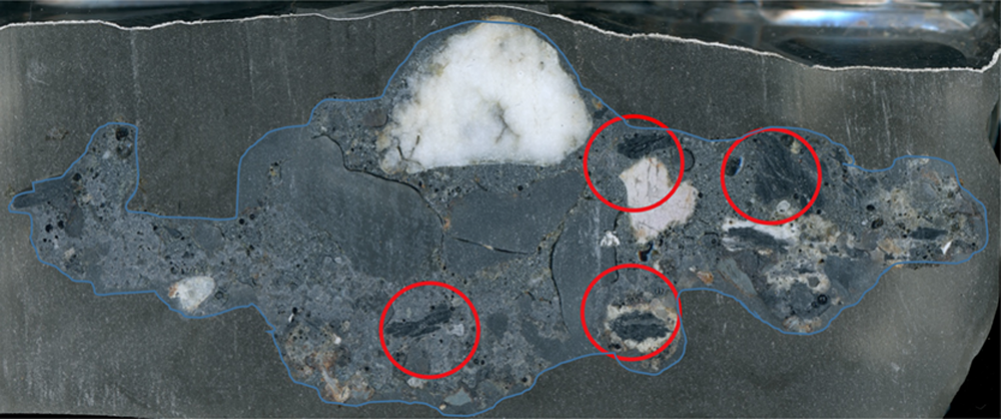
Cross section of ash taken from the Colombian sugar mill stoker furnace. The blue line and shading highlight the outline of the deposit in resin. Red circles highlight the location of carbon-rich lumps encased inside the deposit.

Figure 10 shows a sectioned slice of FBA encased in resin. The red circled areas show the carbon material that is surrounded by ash deposit. The presence of large carbon lumps in bottom furnace ash and the ash deposits on the surface of the chars is likely to be the key to incomplete combustion of the coals. These lumps of carbon have no access to oxygen and hence remain unburned. The lack of oxygen means that this carbon will remain unburnt regardless of the residence time in the furnace. The only real means of “restarting” combustion would be to mechanically break open the lump of ash to expose enclosed carbon surfaces.

While higher bed temperatures or longer residence times would be unlikely to change the overall combustion efficiency, coal beneficiation would certainly lower the overall ash content of the coals and therefore decrease the rate at which carbon materials become enveloped by ash layers.^42^

Based on the TGA profiles and reflectance analysis, Cundinamarca and Valle are the coals that are most likely to be trapped inside the FBA lumps. These coals both have relatively high ash but, more importantly, have high levels of inherent mineral in mid- and large-sized particles (6–19; >19 mm). The −6 mm size range shows a high degree of liberated mineral, but this, in turn, potentially just fills the voidage space on the bed and accelerates the rate at which carbon particles are enveloped by hot ash, further decreasing the availability of oxygen.

## 3 Conclusions

This study presents the application of both established and novel techniques to isolate the causes of poor burnout in Colombian sugar mill stoker furnaces. Petrographic and proximate analysis and other standard tests all indicated that these coals should burn readily in a stoker operating at >900 °C with a residence time of several hours.

While the micro-TGA tests showed that combustion rates were good and complete burnout was achievable during combustion tests, the macro-TGA method showed that burnout was significantly slower and, in some cases, incomplete even when temperatures reached 800 °C. Incomplete combustion was as high as 80% for Cundinamarca for larger particle sizes (>19 mm).

Char samples produced at 700, 900, and 1050 °C and 10, 30, and 120 min were used to recreate materials that were representative of the time temperature history seen in a stoker furnace. Furnace temperature appeared to have the greatest influence on increasing the reflectance and intrinsic reactivity (TGA) of the char, with some chars reaching over 8% reflectance, which is similar to high quality coke. These char reflectance values were correlated with values for ashes from Colombian stoker furnaces. Reflectance values for FBA and fly ash from industrial furnaces were above 6%, indicating that bed temperatures in the stoker furnace were indeed within the expected temperature range to allow complete combustion.

This comparison also allowed Valle and Cundinamarca to be identified as the most likely source of the carbon inside the FBA and fly ash. The high ash content (29.2%) of the −6 mm fraction of the Patia coal probably contributed to the problem through the filling of voidage space with high ash particles. Antioquia, however, was not responsible for the burnout issues as it was a reactive low ash coal (4.5–13%) and was unable to produce carbon with the same properties (reflectance 2.23–5.61) as those seen in the FBA and fly ash (>6%). Even when heated beyond the operating temperature of the stoker (>1000 °C), the reflectance value of Antioquia was only 5.61% after a residence time of 540 min.

Also, 19–55% of the coals were found to be −6 mm, which is below the target specification for particle size, and the fines contained 13–29% ash (dafb). The use of char reflectance as a predictive tool demonstrated that poor oxygen diffusion led to poor burnout as the carbon inside the bed is shielded from oxygen ingress through the formation of nonpermeable ash layers.

Finally, this study demonstrates for the first time how carbon reflectance and intrinsic reactivity can be used as a diagnostic tool for evaluating the thermal history and operational performance of stoker furnaces and, in the future, potentially of boilers.

## 4 Materials and Methods

### 4.1 Colombian Stoker Furnaces and Coal Materials

This study analyzed four Colombian coals used in stoker furnaces in Colombian sugar mills. The coals were all from distinct mining regions around Colombia: Antioquia, Cundinamarca, Patia, and Valle. In addition, fly ash and FBA samples were obtained from stoker furnaces in the Mayagüez sugar mill (37 MW electricity) and La Cabaña sugar mill (30 MW electricity) located in the Valle Del Cauca, Colombia. The coal blend at La Cabaña was 30% Cundinamarca, 30% Antioquia, 30% Valle, and 10% Patia. The target feed size for both La Cabaña and Mayagüez stoker furnaces is specified as 6–20 mm (60%) and <6 mm (40%), respectively.

The residence times for Mayagüez and La Cabaña were 4–6 and 2–2.5 h, respectively. The Mayagüez furnace has a 180 ton steam/h and pressure of 67 bar and 25% excess air for coal. The La Cabaña furnace has a 149 ton steam/h and pressure of 67 bar and 30% excess air. Both furnaces have operating temperature ranges between 800 and 1000 °C and an overheated steam temperature of 510 °C. Fly ash samples were sampled from a fly ash hopper, and bottom ash was sampled from the bottom ash belt after quenching in the water bath.

Stoker furnaces generally operate in a particle size range of ∼6–25 mm^3^, so particle size distribution was determined using the closest available sieves available sieving 10 kg of each dried coal into three size fractions; <6, 6–19, and >19 mm.

### 4.2 Thermal Characterization

#### 4.2.1 Microthermogravimetric Analysis

Thermogravimetric analysis (TGA) was used to analyze the thermal properties of the samples. Thermal profiles were produced using a TA Instruments Q500 TGA (New Castle, DE, USA). TGA tests used 10–15 mg milled to <300 μm. The composition of the samples is given by moisture, dry volatile, fixed carbon, and dry ash contents in accordance with BS ISO 17246:2010.^49^ The intrinsic reactivity of the coals and chars was assessed using a technique described previously.^28^ The weight loss profile was used to obtain the initial temperature, burnout temperature, and peak temperature for each sample where the weight loss increases to 0.2%/min, reached a maximum, and drops to 0.2%/min, respectively.^50^ The 0.2%/min weight loss point was chosen to represent a point combustion profile visibly began, and it allows the temperature range of combustion (initial to burnout) to be quantified. While it is possible to use higher or lower thresholds,^51,52^ it remains an arbitrary, but relative threshold that best describes the combustion profiles seen in this study.

#### 4.2.2 Macrothermogravimetric Analysis

**Figure 11:**
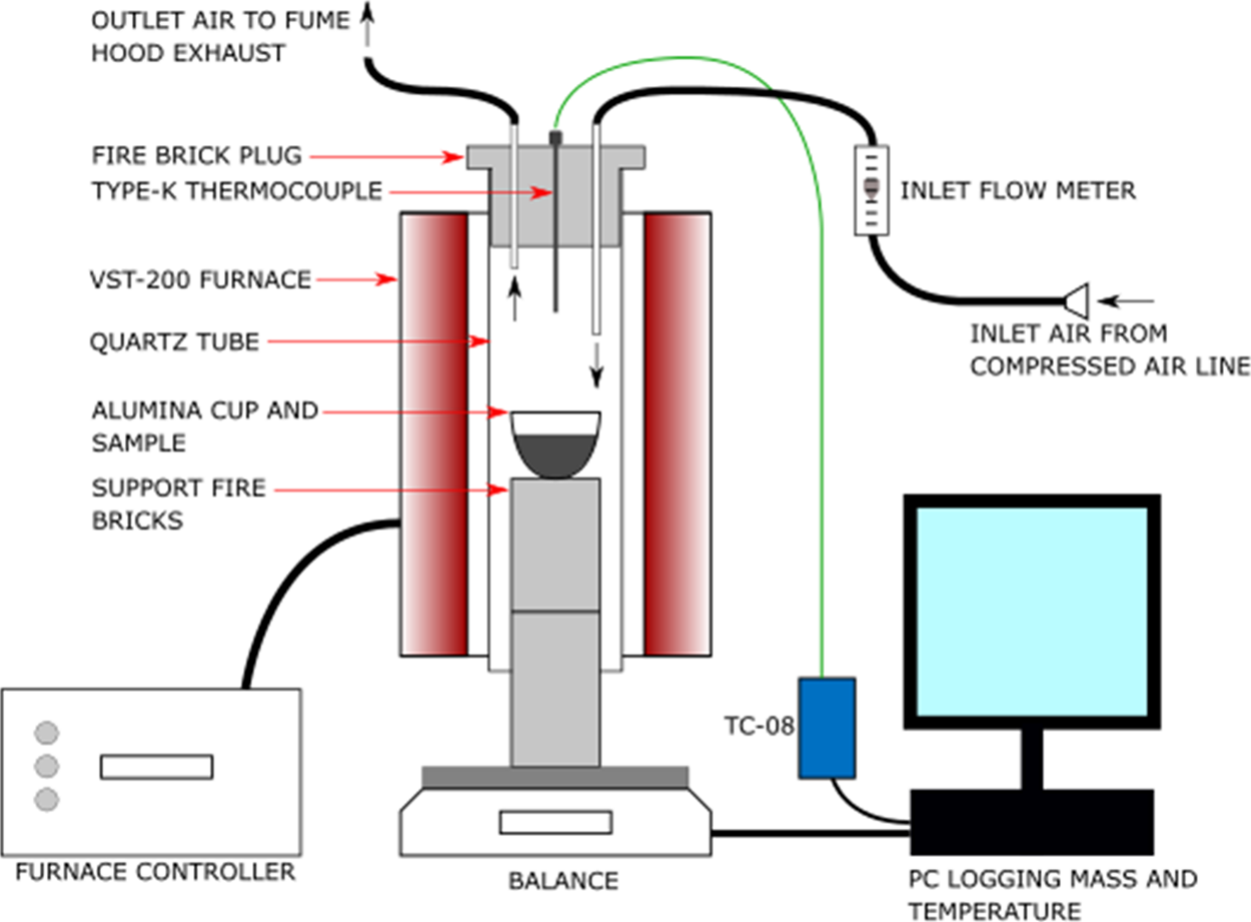
Macro-TGA configuration.

The micro-TGA resulted in the complete combustion of the samples (Figure 1). However, it was known from the samples taken from the furnace (Figure 10) that a significant amount of unburnt carbon was seen in the samples after 2–3 h in the furnace. In order to investigate this further, a novel macroscale TGA (macro-TGA) was constructed for this study as shown in Figure 11. The aim of the system was to replicate the incomplete combustion seen in industry on a laboratory scale and demonstrate that the mass loss was significantly different to that obtained from a micro-TGA.

The macro-TGA was located in a fume hood to contain all exhaust gases. The macro-TGA consisted of a Carbolite VST-200 vertical tube furnace with a 65 mm quartz tube. An Ohaus Pioneer PA4202C balance connected to a PC logged the weight of the sample every second via the Ohaus DAS software. Two cored insulating fire bricks (Victas grade 30) and an Almath alumina crucible were placed on top of the balance and tared. Approximately, 1 g of <6 mm, 4–5 g of 6–19 mm, and 4–9 g of >19 mm of each sample were placed into the Almath crucible and weighed. The crucible was placed on top of the first support fire brick and then raised into the quartz tube, with the second fire brick placed underneath the first so that the crucible was in the middle of the furnace. There was a 1 mm gap between the support fire bricks and the quartz tube. The top of the quartz tube had a fire brick plug with three inserts: a type K 310 stainless steel sheath thermocouple (TC direct), a gas inlet pipe, and an exhaust pipe. The thermocouple was connected to a PicoLogger TC-08 box and logged temperature readings every second on the PicoLog 5.25.3 software.

**Figure 12:**
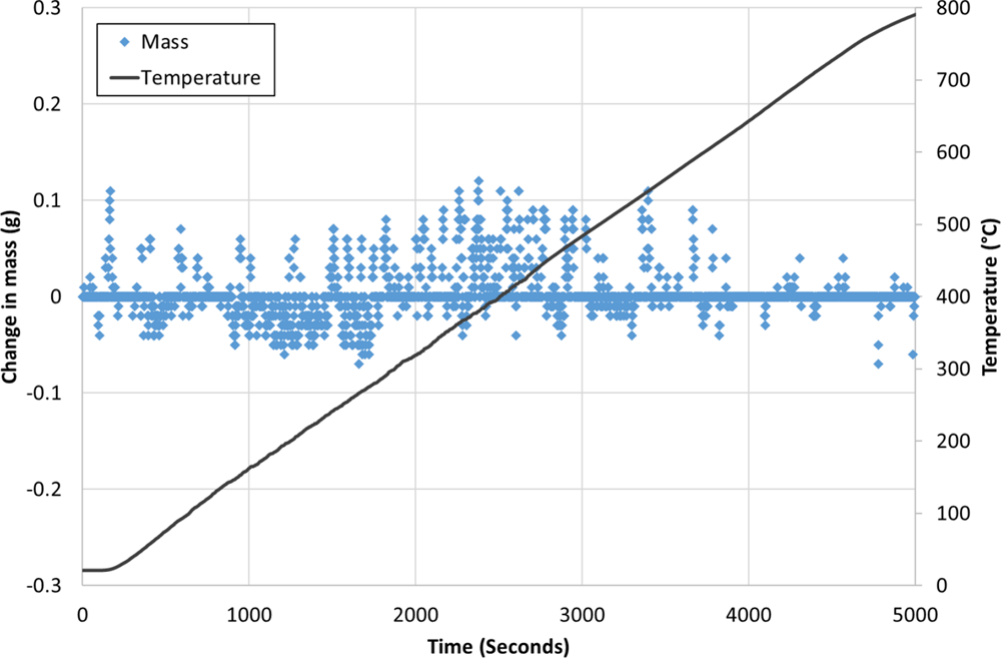
Temperature profile and mass change at a 10 °C/min ramp rate using an empty crucible.

The gas inlet was connected to a compressed air supply from the fume hood and regulated to 5 L/min with a Platon air flow regulator. The air supply was connected to a steel pipe in the fire brick plug at the top of the quartz tube. This steel pipe protruded approximately 5 cm past the base of the fire brick plug to supply air to the furnace. The air inlet was placed to ensure that it was far enough away from the exhaust but not too close to the sample as to cause mass fluctuations. A steel pipe was also used for the exhaust but only protruded slightly from the base of the fire brick plug. This ensured maximum separation between the air inlet and the exhaust. The exhaust steel pipe was connected to a plastic pipe at the top of the fire brick plug, which then routed to the top of the fume hood so that all exhaust gases were evacuated from the space. The furnace was set to ramp at 10 °C.min^–1^ to match the intrinsic reactivity method up to 800 °C. Tests were conducted in duplicate for <6, 6–19, and >19 mm size fractions. A tared empty crucible test was conducted, and this base line data were subtracted from the weight of each run to remove drift in the measurements. Due to the explosive nature of some of the samples during combustion,^36^ weight data were smoothed in Matlab 2019a using the smoothn.m script by Garcia,^53^ which is an automated smoothing procedure for uniformly sampled data sets based on a penalized least squared method, which allows smoothing of the results in one or higher dimensions by means of the discrete cosine function. Figure 12 shows the temperature ramp profile and mass change for an empty tared crucible from atmospheric conditions to 800 °C in the macro-TGA at a ramp rate of 10 °C/min. The average mass change over the test was 0.016 g with a standard deviation of 0.05 g, and the average temperature ramp rate was 9.5 °C/min with a standard deviation of 1.2 °C/min. The linearity deviation on the Ohaus Pioneer balance is typically ±0.06 g up to a maximum of 0.2 g. Thus, the mass fluctuations observed are within the linearity deviation allowed for the balance. A ramp rate of 10 °C/min was selected to match the ramp conditions of the intrinsic reactivity test in the micro-TGA.

#### 4.2.3 Muffle Furnace

In addition, a series of char samples was produced using a Vecstar VF1 muffle furnace oven with a nitrogen supply of 10 L/min. Almath alumina classic crucibles with a lid were used for all experiments to minimize combustion. Tests were conducted on all four coal samples for three particle sizes (<6, 6–19, and >19 mm), three temperatures (700, 900, and 1050 °C), and three residence times (10, 30, and 120 min), totaling 108 tests. The mass of the samples before and after testing was noted, and the samples were characterized by reflectance, elemental, and TGA analysis.

#### 4.2.4 Ultimate Analysis and Higher Heating Value

Ultimate analysis was conducted with a LECO CHN-628 series elemental analyzer for carbon (C), hydrogen (H), and nitrogen (N) according to ASTM 5373.^54^ For the raw coals, sulfur content (S) was determined with a LECO 628 S according to ASTM 4239.^55^ The O content for the muffle furnace chars was calculated by the difference of C, H, and N values from 100% (on a dry ash-free basis). The higher heating values of the samples were found using an IKA C5000 Bomb Calorimeter in accordance with BS ISO 1928:2009.^56^

### 4.3 Mineral Composition

MLA was used to determine the mineral separation and location in coal particles. The samples were prepared in carnauba wax with an epoxy resin backing, ground using 800/1200/2400 grit silicon carbide paper and polished with 6 μm diamond solution. Polished blocks were coated with a 10 nm carbon film using a Quorum Q150T Thin-Film Coater, and MLA was conducted with a scanning electron microscope model FEI Quanta 600i SEM with energy-dispersive X-ray. The FEI MLA 3.1 software was used to relate the mineral content of the coal particle to its surface area.^57^

### 4.4 Density

XRF was carried out by Servicio Geológico Colombiano in Bogotá, Colombia. Samples were ashed and prepared into XRF ready disks and were then analyzed using a Thermo Scientific ARL Perform’X sequential XRF spectrometer using standard protocols.^58^

For coal particles under 6 mm, particle density was obtained using a helium gas pycnometer (AccuPyc II 1340, Micromeritics, UK) with a 1 cm^3^ cell and helium gas at an equilibration rate of 0.005 psig/min for 10 cycles and purges.^59^ For particles between 6 and 19 mm, density was obtained using an Ohaus buoyancy rig on an Ohaus Pioneer 4 point balance.^60^

Tapped density was obtained via a Copley Scientific Series JV tap density tester.^61^ Using a 100 mL measuring cylinder with a 40 g sample of <6 and 6–19 mm and then after 1000 taps, the volume of the sample was noted to provide the tapped density of the sample.

### 4.5 Petrographic Analysis

Reflectance analysis was carried out using polished blocks of the samples (all samples ground to <1 mm), prepared with an epoxy liquid resin blend and examined manually using a polarized-light microscope (Zeiss Leitz Ortholux Pol II BK) with a ×50 magnification oil-immersion objective and ×10 magnification eyepiece.^62^ The random reflectance of each sample was measured using a Leitz spectrophotometer calibrated using a silicon carbide light standard (7.51% reflectance in oil) and zirconia standard (3.17% reflectance in oil).^63^

## Supporting Information Available

The vitrinite reflectance profiles for Cundinamarca (a), Antioquia (b), Patia (c), and Valle (d) in the three size fractions (<6, 6–19, and >19 mm) (Figure S1A–D), micro- and macro-TGA profiles for Antioquia (A), Valle (B), and Patia (C) coals heated at 10 °C.min^–1^ in air (Figure S2), mass loss for varying particle sizes of Antioquia (A), Valle (B), and Patia (C) coals heated for varying temperatures and residence times in a muffle furnace (Figure S3), the reflectance data for the chars prepared in a muffle furnace at 700, 900, and 1050 °C for Antioquia (A), Cundinamarca (B), Patia (C), and Valle(D) (Table S1A–D), average reflectance against temperature for Antioquia, Cundinamarca, Patia, and Valle (Figure S4), average reflectance against time (minutes) for Antioquia, Cundinamarca, Patia, and Valle (Figure S5), the intrinsic reactivity data (initial (A), peak (B), and burnout (C) temperatures) for the 18 char samples prepared at 10, 30, and 120 min at 700, 900, and 1050 °C (Table S2A–C), MLA analysis of (A) Antioquia 6–19 mm, (B) Cundinamarca 6–19 mm, (C) Valle 6–19 mm, and (D) Patia 6–19 mm (Figure S6A–D), MLA analysis of (A) Antioquia >19 mm, (B) Cundinamarca >19 mm, (C) Valle >19 mm, and (D) Patia > 19 mm (Figure S7A–D), MLA data for Antioquia and Cundinamarca (Table S3), MLA data for Patia and Valle (Table S4), and XRF data for coals and chars (Table S5) (PDF)
