# Supplementary material for: Novel Thermal and Microscopic Techniques To Determine the Causes of Suboptimal Combustion Performance at Colombian Stoker Furnaces
Source: ACS Omega. 2022 Apr 2;7(14):11618–30. doi: 10.1021/acsomega.1c06314 (PMC9017112; doi:10.1021/acsomega.1c06314)
Supplement: Supplementary file 1 — ao1c06314_si_001.pdf [file ao1c06314_si_001.pdf]

Novel thermal and microscopic techniques to determine the causes of sub-optimal combustion performance at Colombian Stoker Furnaces

Orla Sioned Aine Williams[1*], Patrick Daley[1], Joseph Perkins[2], Shoaib Shah[1], Edward Andres Garcia Saavedra[3], Maria Trujillo[3], Juan Barraza-Burgos[3], Carlos Julio Espitia[4], Maribel Barajas[4], Juan Sebastian Saltaren[5], Nicolás Javier Gil[6], Edward Henry Lester[1]

[1]*Faculty of Engineering, University of Nottingham, University Park, Nottingham, NG7 2RD, United Kingdom*

[2]*Mineral Resources, Commonwealth Scientific and Industrial Research Organisation, 1 Technology Court, Pullenvale QLD 4069, Australia*

[3]*Facultad de Ingeniería, Universidad Del Valle, Ciudad Universitaria Meléndez, Calle 13 # 100-00. A. A., Cali, 439, Colombia*

[4]*Servicio Geológico Colombiano, Diagonal 53 N0. 34 – 53, Bogotá D.C. 11121, Colombia*

[5]*Grupo Manuelita, Calle 6 # 3 - 13, Cali, 760044, Colombia*

[6]*Centro de Investigacion de la Cana de Azucar de Colombia, Programa de procesos de fábrica, calle 58 Norte No 3BN.110,Cali, CO 780001, Colombia*

**orla.williams@nottingham.ac.uk* *+44 115 748 6854*

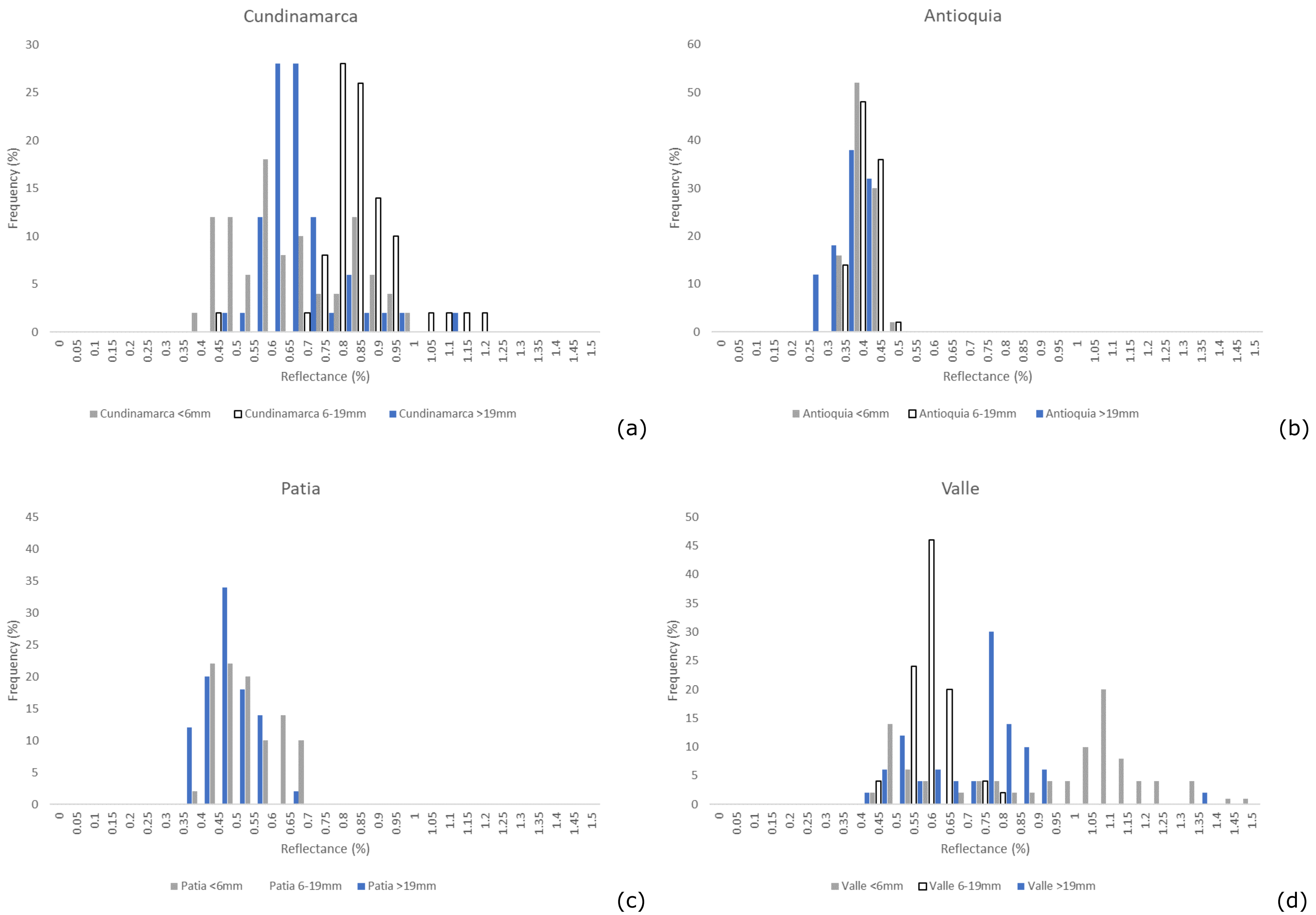

Figure S1a-d – The vitrinite reflectance profiles for Cundinamarca (a), Antioquia (b), Patia (c), and Valle (d) in the three size fractions (<6mm, 6-19mm, >19mm)

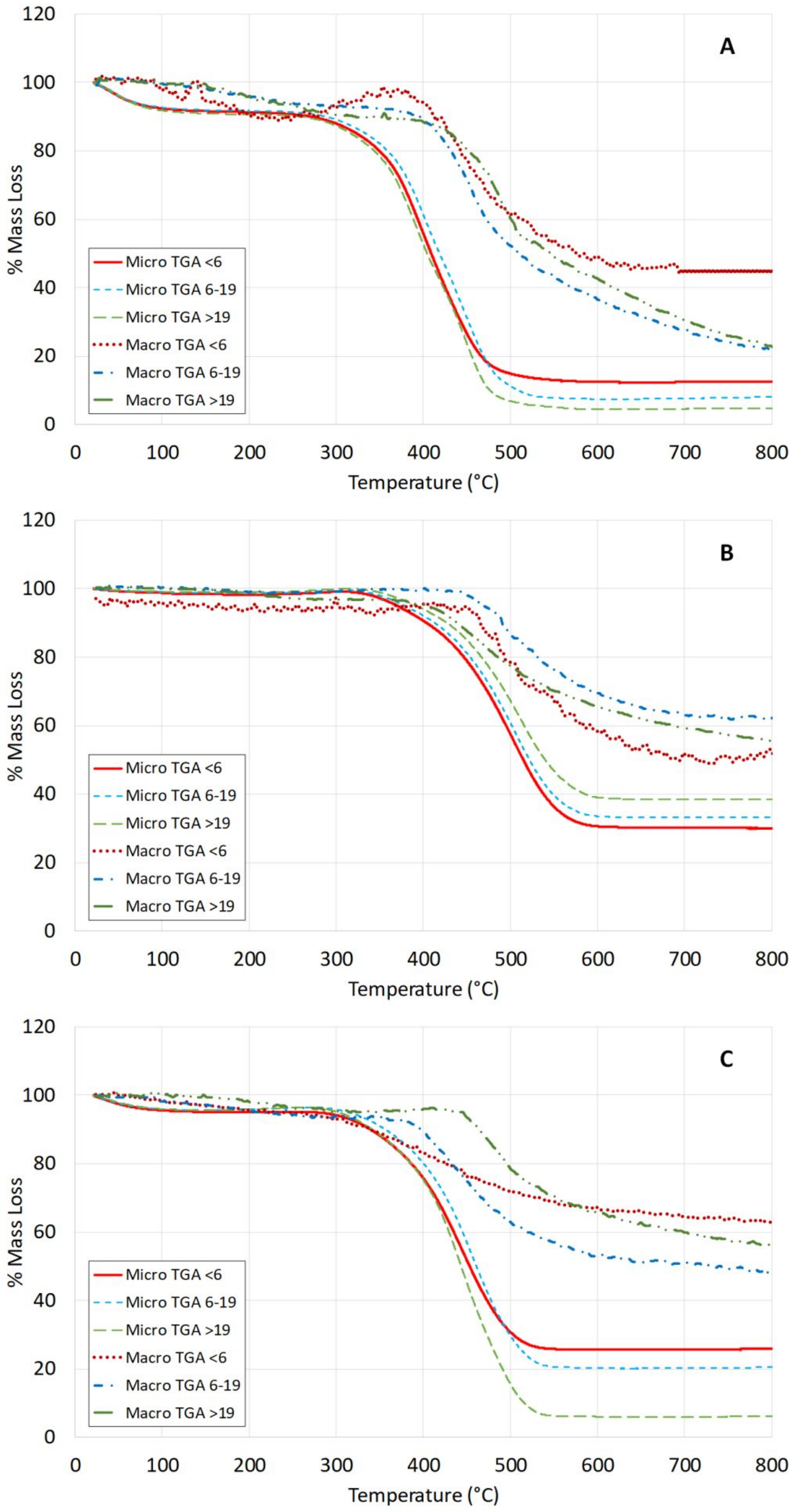

Figure S2 – Micro and Macro TGA profiles for Antioquia (A), Valle (B) and Patia (C) coals heated at 10 °C.min^{-1} in air

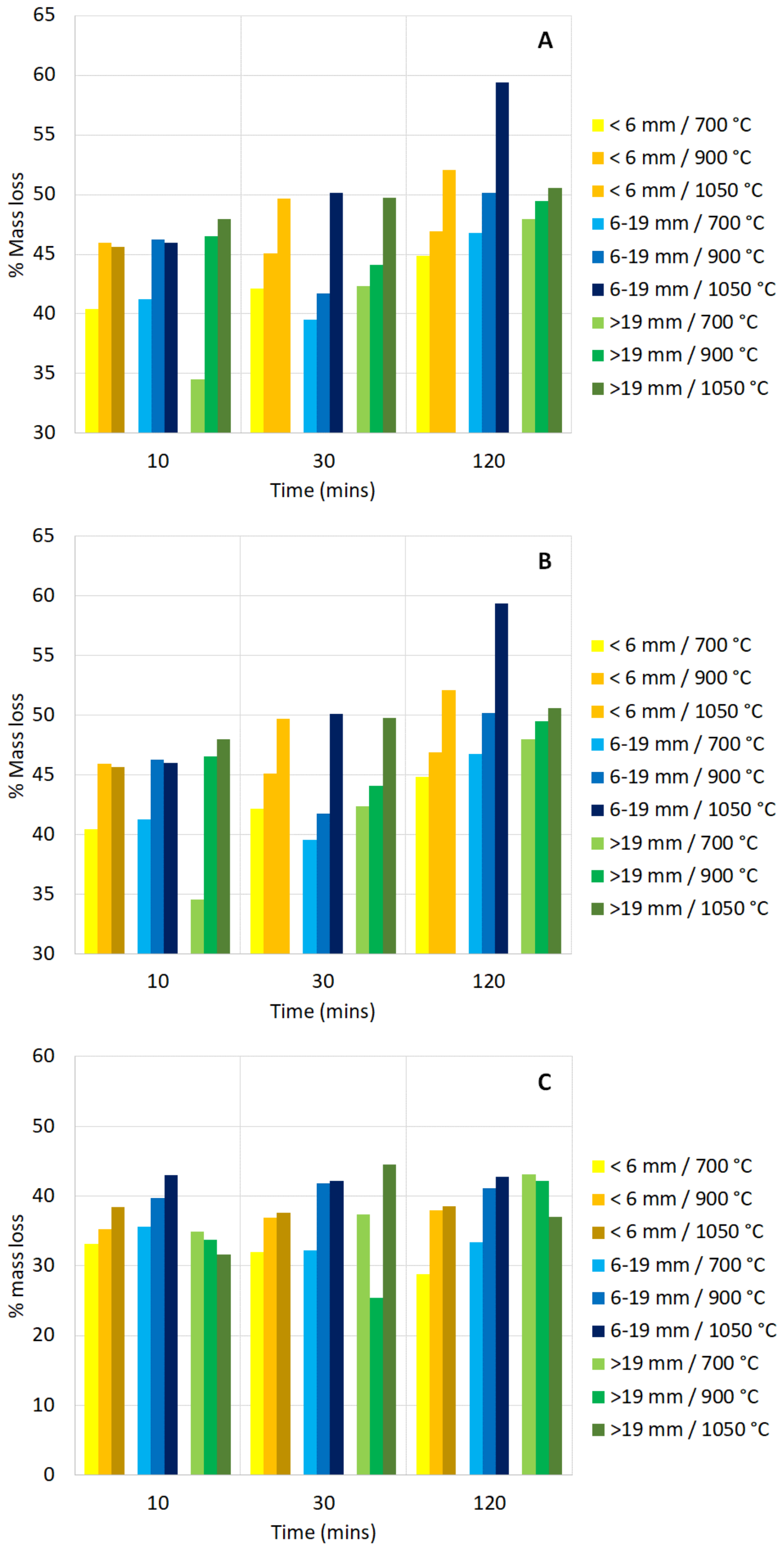

Figure S3 – Mass loss for varying particle sizes of Antioquia (A), Valle (B) and Patia (C) coals heated for varying temperatures and residence times in a muffle furnace

	(A)	Antioquia								
		<6mm			6-19mm			>19mm		
700ºC		*10 mins*	*30 mins*	*120 mins*	*10 mins*	*30 mins*	*120 mins*	*10 mins*	*30 mins*	*120 mins*
	**Average**	**2.15**	**2.77**	**3.78**	**2.09**	**2.83**	**4.15**	**2.45**	**2.87**	**3.94**
	Minimum	1.80	2.27	2.65	1.56	2.16	3.42	1.73	2.09	3.04
	Maximum	2.56	3.17	4.76	2.56	3.36	4.75	2.92	3.36	4.59
	St.Dev.	0.183	0.248	0.491	0.221	0.300	0.361	0.245	0.278	0.362
900ºC		*10 mins*	*30 mins*	*120 mins*	*10 mins*	*30 mins*	*120 mins*	*10 mins*	*30 mins*	*120 mins*
	**Average**	**4.05**	**4.43**	**4.70**	**4.35**	**4.75**	**4.91**	**4.43**	**4.60**	**4.97**
	Minimum	2.57	2.35	3.12	2.86	3.40	3.50	3.39	3.28	3.71
	Maximum	5.19	6.29	5.72	5.48	5.82	6.11	5.50	5.63	5.96
	St.Dev.	0.747	0.827	0.503	0.759	0.676	0.687	0.605	0.571	0.503
1050ºC		*10 mins*	*30 mins*	*120 mins*	*10 mins*	*30 mins*	*120 mins*	*10 mins*	*30 mins*	*120 mins*
	**Average**	**4.95**	**5.08**	**5.14**	**5.03**	**5.31**	**5.52**	**4.77**	**4.86**	**5.36**
	Minimum	4.00	3.97	4.02	3.41	4.04	4.35	3.03	3.55	4.05
	Maximum	6.13	5.95	5.77	6.21	6.36	6.71	5.84	5.63	6.83
	St.Dev.	0.546	0.508	0.439	0.691	0.644	0.394	0.721	0.519	0.642

(B)		Cundinamarca <6mm			6-19mm			>19mm		
		10 mins	*30 mins*	*120 mins*	*10 mins*	*30 mins*	*120 mins*	*10 mins*	*30 mins*	*120 mins*
700ºC	**Average**	**2.03**	**2.64**	**3.79**	**2.53**	**3.71**	**4.40**	**2.35**	**3.93**	**4.35**
	Minimum	1.58	1.83	1.86	1.71	2.51	3.13	1.81	2.49	1.54
	Maximum	3.02	3.55	5.98	3.37	5.45	5.36	3.84	5.21	6.92
	St.Dev.	0.275	0.415	0.815	0.363	0.578	0.551	0.394	0.622	1.228
		10 mins	*30 mins*	*120 mins*	*10 mins*	*30 mins*	*120 mins*	*10 mins*	*30 mins*	*120 mins*
900ºC	**Average**	**5.03**	**5.35**	**5.47**	**5.04**	**5.44**	**5.69**	**5.07**	**5.72**	**6.23**
	Minimum	3.12	3.70	4.00	3.74	4.03	4.14	3.10	3.47	4.11
	Maximum	7.26	6.82	7.37	7.85	6.77	7.56	6.55	6.80	7.82
	St.Dev.	1.001	0.745	0.777	0.806	0.628	0.826	0.693	0.707	0.894
		10 mins	*30 mins*	*120 mins*	*10 mins*	*30 mins*	*120 mins*	*10 mins*	*30 mins*	*120 mins*
1050ºC	**Average**	**6.02**	**6.69**	**7.06**	**5.76**	**6.43**	**6.47**	**6.04**	**6.42**	**6.65**
	Minimum	4.35	4.92	5.39	3.92	3.70	5.01	4.12	4.78	5.16
	Maximum	7.84	8.32	8.67	8.91	9.55	8.34	8.67	8.60	8.51
	St.Dev.	0.880	0.887	0.906	1.093	1.297	0.837	0.969	0.991	0.735

		Patia								
	**(C)**	<6mm			6-19mm			>19mm		
		10 mins	*30 mins*	*120 mins*	*10 mins*	*30 mins*	*120 mins*	*10 mins*	*30 mins*	*120 mins*
	**Average**	**2.42**	**2.89**	**3.29**	**2.03**	**2.22**	**3.39**	**2.55**	**2.94**	**3.59**
700°C	Minimum	1.71	2.01	1.66	1.57	1.82	2.37	1.94	2.37	2.80
	Maximum	2.80	3.40	4.32	2.47	2.73	4.03	3.03	3.59	4.34
	St.Dev.	0.268	0.283	0.725	0.243	0.172	0.440	0.248	0.283	0.390
		10 mins	*30 mins*	*120 mins*	*10 mins*	*30 mins*	*120 mins*	*10 mins*	*30 mins*	*120 mins*
	**Average**	**4.54**	**4.74**	**4.75**	**4.38**	**4.53**	**4.81**	**4.61**	**4.76**	**4.92**
900°C	Minimum	3.39	3.52	3.34	3.38	3.52	3.32	3.05	3.62	3.07
	Maximum	5.49	5.55	6.06	5.44	5.28	5.60	5.79	5.70	5.97
	St.Dev.	0.450	0.522	0.649	0.621	0.391	0.525	0.653	0.626	0.736
		10 mins	*30 mins*	*120 mins*	*10 mins*	*30 mins*	*120 mins*	*10 mins*	*30 mins*	*120 mins*
	**Average**	**5.10**	**5.29**	**5.47**	**5.21**	**5.53**	**5.85**	**5.04**	**5.48**	**5.81**
1050°C	Minimum	4.02	3.47	4.32	3.98	4.20	3.63	3.86	4.47	3.77
	Maximum	5.91	6.48	6.42	6.32	6.09	7.66	5.85	6.61	9.93
	St.Dev.	0.458	0.862	0.501	0.573	0.474	0.763	0.533	0.535	1.205

		Valle								
	**(D)**	<6mm			6-19mm			>19mm		
$700^{o}C$		*10 mins*	*30 mins*	*120 mins*	*10 mins*	*30 mins*	*120 mins*	*10 mins*	*30 mins*	*120 mins*
	**Average**	**2.01**	**3.91**	**4.24**	**2.32**	**4.10**	**4.58**	**2.51**	**3.12**	**5.13**
	Minimum	1.50	2.53	2.29	1.69	2.73	2.31	1.51	2.33	3.34
	Maximum	3.18	6.35	7.06	4.67	6.11	8.00	3.28	3.88	8.37
	St.Dev.	0.343	0.898	0.856	0.507	0.859	1.215	0.351	0.365	0.981
$900^{o}C$		*10 mins*	*30 mins*	*120 mins*	*10 mins*	*30 mins*	*120 mins*	*10 mins*	*30 mins*	*120 mins*
	**Average**	**5.07**	**5.53**	**5.93**	**5.39**	**5.92**	**6.14**	**5.85**	**6.12**	**6.37**
	Minimum	2.49	4.00	4.09	3.75	4.22	4.06	3.91	3.91	3.37
	Maximum	8.48	9.03	8.75	10.07	9.39	9.73	9.66	9.87	10.42
	St.Dev.	1.517	1.107	0.923	1.476	0.898	1.249	1.130	1.431	1.764
$1050^{o}C$		*10 mins*	*30 mins*	*120 mins*	*10 mins*	*30 mins*	*120 mins*	*10 mins*	*30 mins*	*120 mins*
	**Average**	**6.20**	**6.53**	**6.50**	**6.12**	**6.70**	**6.69**	**6.30**	**6.63**	**6.64**
	Minimum	4.39	4.98	4.57	3.46	4.63	4.37	4.02	4.53	5.13
	Maximum	8.83	10.06	7.99	9.00	9.09	10.18	9.90	9.78	9.69
	St.Dev.	1.261	1.238	0.682	1.244	1.200	1.564	1.454	1.233	1.165

Table S1 (A)-(D) – the reflectance data for the chars prepared in a muffle furnace at $700^{o}C$, $900^{o}C$ and $1050^{o}C$ for Antioquia (A), Cundinamarca (B), Patia (C) and Valle (D)

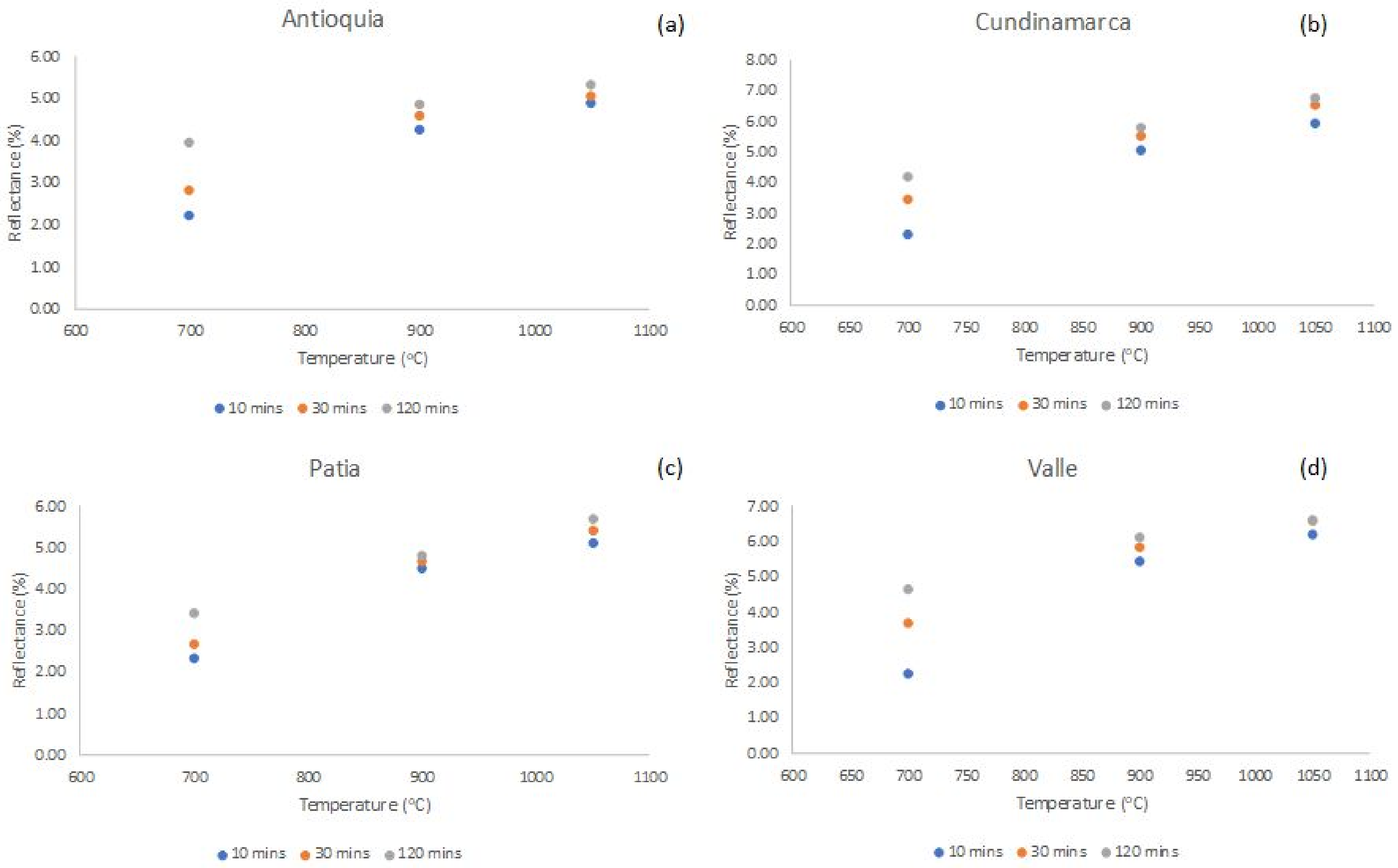

Figure S4 – Average reflectance against temperature for Antioquia (a) , Cundinamarca (b), Patia (c) and Valle (d)

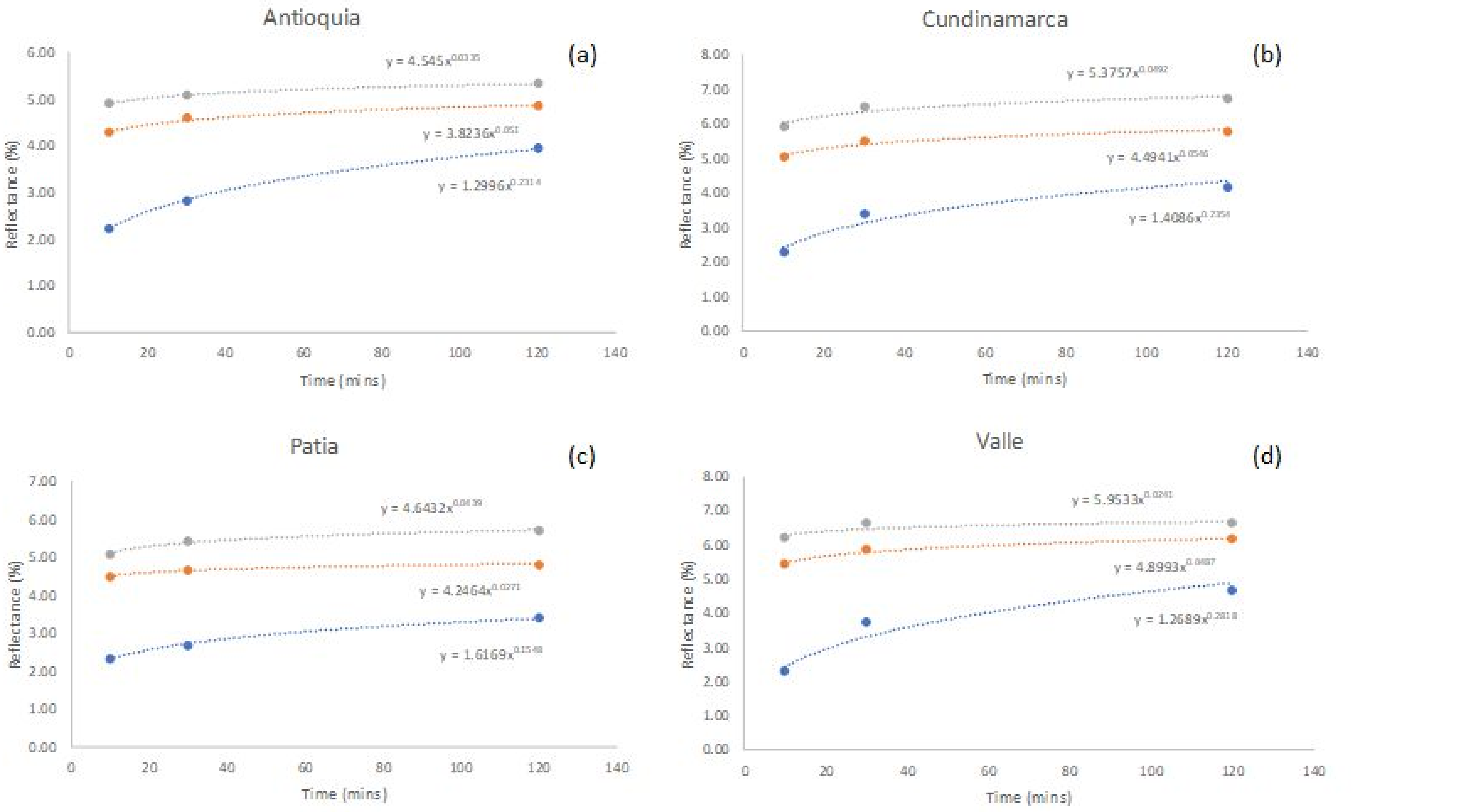

Figure S5 – Average reflectance against time (minutes) for Antioquia (a), Cundinamarca (b), Patia (c) and Valle (d)

(A) INITIAL TEMPERATURE (°C)	Time (mins)	Antioquia Size (mm)			Cundinamarca Size (mm)			Patia Size (mm)			Valle Size (mm)		
		<6	6-19	>19	<6	6-19	>19	<6	6-19	>19	<6	6-19	>19
700	10	306	303	333	346	387	434	336	332	369	368	394	371
	30	316	330	334	348	400	424	370	363	380	423	437	448
	120	363	335	331	386	405	407	388	372	381	427	441	430
900	10	318	320	376	371	446	431	401	391	403	424	446	461
	30	354	360	362	418	461	431	402	393	416	417	447	451
	120	383	365	379	420	457	434	408	471	417	446	446	425
1050	10	392	379	402	465	475	501	396	461	464	447	473	491
	30	410	380	403	470	474	510	451	442	465	447	471	495
	120	424	383	402	501	532	559	456	463	521	546	529	550

(B) PEAK TEMPERATURE (°C)	Time (mins)	Antioquia Size (mm)			Cundinamarca Size (mm)			Patia Size (mm)			Valle Size (mm)		
		<6	6-19	>19	<6	6-19	>19	<6	6-19	>19	<6	6-19	>19
700	10	477	508	578	598	623	625	547	551	561	599	612	603
	30	548	539	580	606	622	712	566	582	585	662	645	623
	120	637	579	624	613	638	624	574	603	574	721	652	662
900	10	544	542	546	642	676	746	581	616	610	639	702	693
	30	601	696	604	681	731	735	601	604	626	683	716	696
	120	611	620	655	701	771	761	628	637	657	755	744	725
1050	10	624	644	653	732	743	794	691	676	673	711	716	785
	30	657	621	682	733	792	812	702	689	673	746	786	800

	120	654	638	718	820	847	835	746	726	744	809	837	820

**(C) BURNOUT TEMPERATURE**		Antioquia			Cundinamarca			Patia			Valle		
	Time	Size (mm)			Size (mm)			Size (mm)			Size (mm)		
(^{o}C)	(mins)	<6	6-19	>19	<6	6-19	>19	<6	6-19	>19	<6	6-19	>19
700	10	732	720	764	774	796	761	720	729	701	796	816	768
	30	767	711	798	806	823	851	758	753	737	832	818	786
	120	793	788	801	792	831	>900	781	771	861	868	814	816
900	10	793	789	716	851	871	>900	740	744	767	801	829	822
	30	816	807	772	853	>900	>900	772	761	791	813	856	824
	120	833	822	831	884	>900	>900	855	821	824	891	887	882
1050	10	803	833	845	>900	>900	>900	801	846	767	>900	>900	>900
	30	856	796	895	>900	>900	>900	852	864	844	>900	>900	>900
	120	841	831	897	>900	>900	>900	>900	>900	878	>900	>900	>900

Table S2A-C – The intrinsic reactivity data (initial (A), peak (B) and burnout (C) temperatures) for the 18 char samples prepared at 10, 30 and 120 minutes at 700°C, 900°C and 1050°C

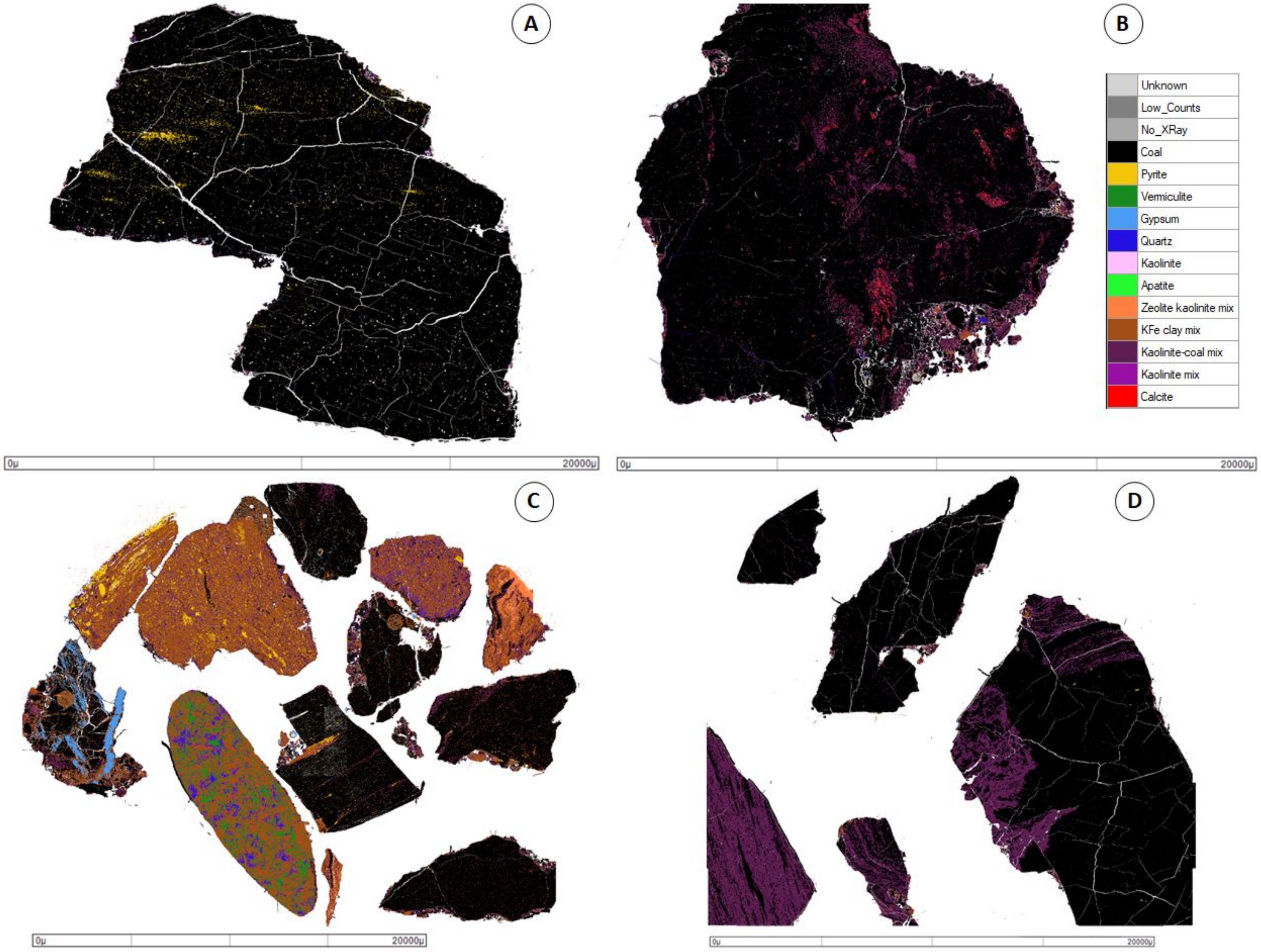

Figure S6A-D – *MLA analysis of (A) Antioquia 6-19 mm, (B) Cundinamarca 6-19 mm , (C) Valle 6-19 mm and (D) Patia 6-19 mm*

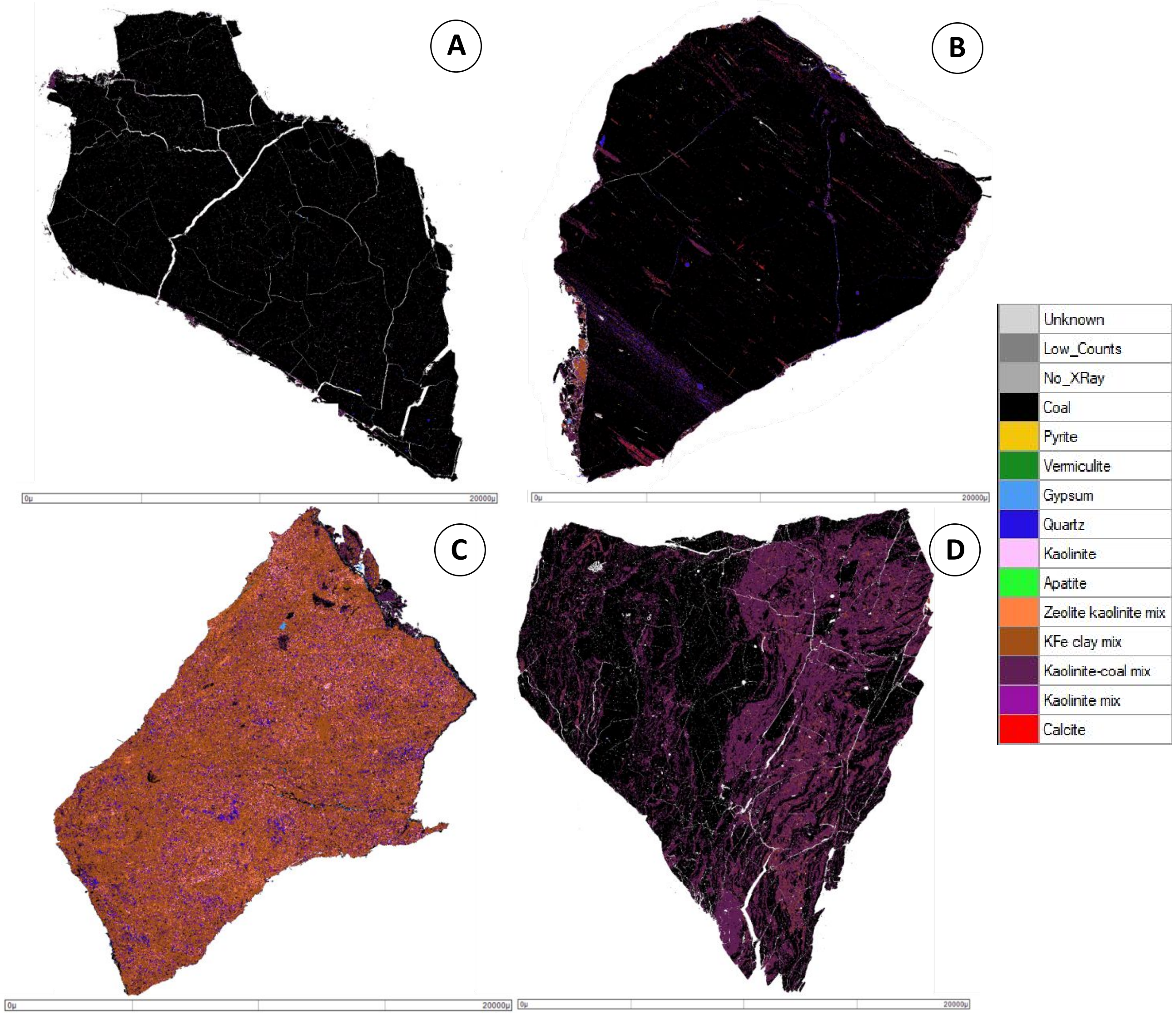

Figure S7A-D – *MLA analysis of (A) Antioquia >19 mm, (B) Cundinamarca >19 mm, (C) Valle >19 mm and (D) Patia >19 mm*

Table S3 – MLA data for Antioquia and Cundinamarca

Mineral	ANTIOQUIA						CUNDINAMARCA					
	<6mm		6-19mm		>19mm		<6mm		6-19mm		>19mm	
	AREA %	WEIGHT %	AREA %	WEIGHT %	AREA %	WEIGHT %	AREA %	WEIGHT %	AREA %	WEIGHT %	AREA %	WEIGHT %
Unknown	0.0%	0.0%	0.0%	0.0%	0.0%	0.0%	0.3%	0.0%	0.3%	0.0%	0.1%	0.0%
Coal	89.0%	88.8%	96.8%	95.6%	98.6%	98.5%	77.2%	76.7%	84.1%	83.5%	93.2%	93.0%
Pyrite	0.1%	0.2%	0.8%	1.9%	0.0%	0.0%	0.0%	0.0%	0.0%	0.1%	0.0%	0.1%
Vermiculite	0.0%	0.0%	0.0%	0.0%	0.0%	0.0%	0.0%	0.0%	0.0%	0.0%	0.0%	0.0%
Gypsum	0.1%	0.1%	0.1%	0.1%	0.1%	0.1%	0.0%	0.0%	0.0%	0.0%	0.0%	0.0%
Quartz	0.0%	0.1%	0.0%	0.0%	0.1%	0.1%	1.2%	1.5%	1.5%	1.9%	0.6%	0.8%
Kaolinite	0.1%	0.2%	0.0%	0.0%	0.0%	0.0%	0.0%	0.0%	0.5%	0.6%	0.0%	0.0%
Apatite	0.0%	0.0%	0.0%	0.0%	0.0%	0.0%	0.0%	0.0%	0.0%	0.0%	0.0%	0.0%
Zeolite kaolinite mix	0.2%	0.2%	0.0%	0.0%	0.0%	0.0%	0.6%	0.5%	0.4%	0.3%	0.3%	0.2%
KFe clay mix	5.0%	4.9%	0.0%	0.0%	0.0%	0.0%	1.4%	1.4%	1.0%	1.0%	0.8%	0.8%
Kaolinite-coal mix	5.2%	5.4%	2.2%	2.3%	1.3%	1.4%	19.1%	19.7%	12.2%	12.5%	4.8%	5.0%

Table S4 – MLA data for Patia and Valle

Mineral	PATIA						VALLE					
	<6mm		6-19mm		>19mm		<6mm		6-19mm		>19mm	
	AREA %	WEIGHT %	AREA %	WEIGHT %	AREA %	WEIGHT %	AREA %	WEIGHT %	AREA %	WEIGHT %	AREA %	WEIGHT %
Unknown	0.0%	0.0%	0.0%	0.0%	0.8%	0.0%	0.0%	0.0%	0.0%	0.0%	0.0%	0.0%
Coal	60.4%	60.2%	75.3%	74.6%	61.7%	61.4%	50.0%	48.9%	38.8%	37.4%	6.8%	6.8%
Pyrite	0.1%	0.2%	0.0%	0.1%	0.0%	0.0%	1.9%	4.6%	1.9%	4.5%	0.0%	0.1%
Vermiculite	0.0%	0.0%	0.0%	0.0%	0.0%	0.0%	0.0%	0.0%	2.7%	3.2%	0.0%	0.0%
Gypsum	0.1%	0.1%	0.0%	0.0%	0.0%	0.0%	0.2%	0.2%	1.4%	1.5%	0.2%	0.2%
Quartz	0.1%	0.1%	0.0%	0.0%	0.0%	0.0%	2.1%	2.7%	2.5%	3.2%	4.1%	5.3%
Kaolinite	0.1%	0.1%	0.0%	0.0%	0.0%	0.0%	0.1%	0.1%	0.1%	0.2%	3.3%	4.2%
Apatite	0.0%	0.0%	0.0%	0.0%	0.0%	0.0%	0.0%	0.0%	0.0%	0.0%	0.0%	0.0%
Zeolite kaolinite mix	0.1%	0.1%	0.0%	0.0%	0.0%	0.0%	3.9%	3.3%	1.0%	0.9%	9.5%	8.3%
KFe clay mix	16.2%	15.9%	0.5%	0.5%	2.5%	2.4%	36.3%	35.0%	42.1%	40.0%	57.5%	56.7%
Kaolinite-coal mix	20.3%	21.0%	24.1%	24.7%	35.1%	36.1%	2.9%	2.9%	8.3%	8.3%	11.0%	11.4%

Table S5 – XRF data for coals and chars

Sample	Silicon Dioxide (wt%)	Titanium Dioxide (wt%)	Aluminium Oxide (wt%)	Iron(III) Oxide (wt%)	Magnesium Oxide (wt%)	Calcium Oxide (wt%)	Sodium Oxide (wt%)	Potassium Oxide (wt%)	Phosphorous Pentoxide (wt%)	Manganese ppm	Vanadium ppm	Strontium ppm	Zirconium ppm
Cundina-marca	64.28	1.24	23.36	6.32	0.75	0.75	0.27	1.56	0.419	179	235	430	317
Antioquia	44.66	1.20	27.52	11.10	2.86	8.87	2.02	1.39	0.071	469	799	432	26
Valle	52.64	1.97	29.29	9.42	1.53	2.03	0.31	0.53	0.051	265	627	199	111
Patia	49.27	1.65	33.59	6.98	1.53	1.99	0.37	1.66	0.108	119	723	326	182
La Cabaña Fly ash	66.72	0.79	17.43	6.52	1.63	2.73	0.79	1.92	0.537	730	188	427	172
La Cabaña Bottom Furnace Ash	59.17	1.21	25.52	7.29	1.44	2.51	0.44	1.47	0.284	345	283	501	199
La Cabaña Char 1	61.29	1.10	25.82	6.39	0.99	1.9	0.15	1.47	0.281	258	240	507	214
La Cabaña Char 2	60.55	1.20	22.78	10.00	1.33	1.59	0.17	1.44	0.191	603	246	263	196
Mayagüez Fly ash	65.83	0.78	15.47	6.95	2.82	3.53	1.40	1.81	0.494	847	154	315	120
Mayagüez Furnace Bottom Ash	68.05	1.20	22.27	4.34	0.88	0.69	< 0.11	1.81	0.196	205	200	366	315
